# The Cytoplasmic Domain of Varicella-Zoster Virus Glycoprotein H Regulates Syncytia Formation and Skin Pathogenesis

**DOI:** 10.1371/journal.ppat.1004173

**Published:** 2014-05-29

**Authors:** Edward Yang, Ann M. Arvin, Stefan L. Oliver

**Affiliations:** Departments of Pediatrics and Microbiology & Immunology, Stanford University School of Medicine, Stanford, California, United States of America; University of Alabama at Birmingham, United States of America

## Abstract

The conserved herpesvirus fusion complex consists of glycoproteins gB, gH, and gL which is critical for virion envelope fusion with the cell membrane during entry. For Varicella Zoster Virus (VZV), the complex is necessary for cell-cell fusion and presumed to mediate entry. VZV causes syncytia formation via cell-cell fusion in skin and in sensory ganglia during VZV reactivation, leading to neuronal damage, a potential contributory factor for the debilitating condition of postherpetic neuralgia. The gH cytoplasmic domain (gHcyt) is linked to the regulation of gB/gH-gL-mediated cell fusion as demonstrated by increased cell fusion *in vitro* by an eight amino acid (aa834-841) truncation of the gHcyt. The gHcyt regulation was identified to be dependent on the physical presence of the domain, and not of specific motifs or biochemical properties as substitution of aa834-841 with V5, cMyc, and hydrophobic or hydrophilic sequences did not affect fusion. The importance of the gHcyt length was corroborated by stepwise deletions of aa834-841 causing incremental increases in cell fusion, independent of gH surface expression and endocytosis. Consistent with the fusion assay, truncating the gHcyt in the viral genome caused exaggerated syncytia formation and significant reduction in viral titers. Importantly, infection of human skin xenografts in SCID mice was severely impaired by the truncation while maintaining the gHcyt length with the V5 substitution preserved typical replication *in vitro* and in skin. A role for the gHcyt in modulating the functions of the gB cytoplasmic domain (gBcyt) is proposed as the gHcyt truncation substantially enhanced cell fusion in the presence of the gB[Y881F] mutation. The significant reduction in skin infection caused by hyperfusogenic mutations in either the gHcyt or gBcyt demonstrates that both domains are critical for regulating syncytia formation and failure to control cell fusion, rather than enhancing viral spread, is severely detrimental to VZV pathogenesis.

## Author Summary

Varicella zoster virus (VZV) infects the human population globally, causing chickenpox in children and shingles in adults. While those afflicted with shingles experience severe pain that might last from weeks to months, the cause is not known. Biopsies of VZV infected skin and specimens of nerve ganglia collected at autopsy from patients with shingles at the time of death contain multi-nucleated cells, indicating that the virus is able to cause fusion between infected cells. Since the destruction of nerve cells that results from this process is likely to contribute to the pain associated with shingles, it is important to understand how the virus causes infected cells to fuse. We find that VZV cell-cell fusion is regulated by the intracellular facing domain of glycoprotein H (gH), a viral protein present on the surface of infected cells. This regulation was dependent upon the physical length of the domain, not a specific sequence. Loss of this regulation increased cell-cell fusion causing the formation of larger multi-nucleated cells that limited the ability of the virus to effectively spread in human skin. Our study provides new insight into how VZV manipulates host cells during infection and controls the spread of the virus in tissues.

## Introduction

Varicella Zoster Virus (VZV) is a ubiquitous human pathogen that causes varicella (chickenpox) in children and zoster (shingles) in adults [1]. Primary infection with VZV initiates at the mucosal epithelium following contact with respiratory droplets or skin vesicle fluid from infected individuals [2]. Viral dissemination in the host occurs by T cell-associated viremia resulting in the infection of skin cells, formation of lesions (chickenpox), and the establishment of latency in neurons of sensory nerve ganglia [3]. Reactivation of VZV from latently infected neurons causes shingles, potentially leading to postherpetic neuralgia (PHN), a condition characterized by severe pain that can last from days to months and in rare cases, for years [4], [5].

Entry of enveloped viruses, including herpesviruses, into a host cell requires fusion of the virion envelope with the host cell membrane [6]. Some enveloped RNA viruses, such as respiratory syncytial virus and DNA viruses, also induce fusion of cell membranes between the infected cells resulting in the formation of a multi-nucleated cell called a syncytium [7], [8]. For VZV, syncytia formation is a hallmark of infection observed in skin lesions as well as trigeminal ganglia taken from cadavers when the individual had zoster at the time of death [9], [10]. Fusion between neurons and their satellite cells in ganglia has been postulated to contribute to the extensive damage caused by VZV reactivation in sensory nerve ganglia and to be a factor for PHN [11]. Mechanisms that regulate VZV syncytia formation can be assessed in cultured melanoma cells and examined for their role in pathogenesis using the human skin and dorsal root ganglia xenografts in the severe combined immunodeficiency (SCID) mouse model [11], [12].

The minimal herpesvirus proteins required for fusion have been determined using virus-free assays that utilize cell-cell fusion as a surrogate for virion envelope and cell membrane fusion. The requirements for fusion *in vitro* differ among the herpesviruses but consist of a core set of glycoproteins that includes glycoprotein B (gB) and the heterodimer of glycoproteins H (gH) and L (gL) [13]. Among alphaherpesviruses, transient expression of gB and gH-gL of VZV and pseudorabies virus (PrV) has been demonstrated to be necessary and sufficient for inducing cell fusion in transfected cells [14], [15], with the caveat that the last eight amino acids (834-841) of VZV gH must be removed for the detection of enhanced fusion under these conditions [15]. Other herpesviruses require additional accessory proteins for fusion, such as gD of herpes simplex virus-1 (HSV-1) and gp42 of Epstein Barr Virus for certain cell types [16], [17]. While transient expression of these glycoproteins induces cell fusion *in vitro*, herpesvirus replication does not trigger syncytia formation, with the exception of VZV and a few naturally occurring HSV variants [18], [19]. Given the differences in requirements for herpesvirus fusion and virally induced syncytia formation, it is necessary to define the functional role of the fusion machinery components of specific herpesviruses independently.

Similar to other herpesviruses, the fusion machinery core of VZV consists of gB, gH, and gL, which are expressed from open reading frame (ORF) 31, 37, and 60, respectively [13], [20]. While the crystal structure of the gB/gH-gL fusion complex has not been determined, X-ray crystallography of the individual glycoprotein components of other herpesviruses has provided insight into their function and roles in fusion. The crystal structure of HSV-1 gB determined the ectodomain to have characteristics of a type III fusion protein [21], whereas the crystal structure of HSV-2 heterodimer gH-gL found the ectodomain to have a “boot-like” structure that differed from other known fusogenic proteins [22]. The current model proposes that gB is expressed as a trimer on the virion surface with two internal fusion loops per monomer and acts as the primary fusogen during herpesvirus entry whereas the gH-gL heterodimer facilitates the fusion potential of gB [13]. However, it remains unclear whether or how gB and gH-gL interact with each other during fusion and while HSV gB/gH-gL complexes have been identified in infected and transfected cells [17], [23], it is not certain if they represent the functional fusion unit. Furthermore, the absence of structural data of a pre-fusion gB form to complement the post-fusion model of gB has limited the understanding of the changes in gB conformation required to trigger fusion. Nonetheless, VZV gB is presumed to be the primary fusogen and gH, together with gL, are essential components of the complex with all three proteins being critical for viral entry and syncytia formation based upon the proposed model.

VZV gH is essential for replication as shown by deleting ORF37 from the VZV genome and inhibiting VZV infection with antibodies against gH in cultured cells and human skin xenografts *in vivo* [24]–[26]. Initially expressed as a 100 kilodalton (kDa) immature polypeptide, gH forms a heterodimer with gL, which is necessary for its maturation to a 118 or 130 kDa form [20], [27]. gH is processed in the Golgi apparatus and traffics to the cell surface, where it then undergoes endocytosis and returns to the trans-Golgi network (TGN) for incorporation into the virion envelope [28]–[32]. gH is predicted to have a single membrane-spanning hydrophobic region with the N-terminus facing the extracellular space when the protein is expressed on the virion envelope or the infected cell surface and the C-terminus projecting toward the viral capsid or the cytoplasm [27].

Our previous work investigating the role of the VZV gH ectodomain in cell fusion led to the identification of motifs in domain I and III that were important for *in vitro* cell fusion and skin pathogenesis [27]. Other *in vitro* studies of the gHcyt identified the ^835^YNKI^838^ sequence as a functional YXXΦ endocytosis motif that contributes to cell fusion when HeLa cells infected with vaccinia virus (VV) expressing T7 polymerase are cotransfected with gH and gL vectors [33]. However, the role of the gHcyt in VZV syncytia formation during infection and pathogenesis has not been investigated.

In a recent study, we identified an immunoreceptor tyrosine-based inhibition motif (ITIM) in the VZV gB cytoplasmic domain (gBcyt) that has a regulatory role in cell fusion and syncytia formation [34]. Substitution of the tyrosine residue in the ITIM with phenylalanine (Y881F) caused an induction in cell fusion at levels significantly greater than the wildtype when the gB mutant was coexpressed with gH[TL], a gH construct lacking amino acids 834-841, and gL *in vitro*. Recombinant viruses with wildtype gH and the gB[Y881F] mutation caused aggressive syncytia formation and reduced viral spread and replication kinetics. Severe impairment of skin pathogenesis *in vivo* was also observed. How the regulation of fusion by the gBcyt relates to the gHcyt has not been fully explored.

In this study, we report that regulation of VZV syncytia formation by the cytoplasmic domain of gH is critical for skin pathogenesis and depends on the length of the domain, not its specific amino acid sequence. Furthermore, both the length of the gHcyt and the gBcyt ITIM must be preserved to prevent exaggerated syncytia formation and allow for effective propagation of VZV. The cytoplasmic domains of these fusion complex proteins contribute differentially to regulation of VZV induced syncytia formation, with both having essential contributions to skin pathogenesis.

## Materials and Methods

### Ethics statement

Institutional Animal Care and Use Committee (IACUC) review of all research involving animals was performed and procedures were approved by the Stanford University Administrative Panel on Laboratory Animal Care (Protocol ID: 11130). Stanford University complies with all federal and state regulations governing the humane care and use of laboratory animals, including the USDA Animal Welfare Act, and the Stanford University Assurance of Compliance with Public Health Service Policy on Humane Care and Use of Laboratory Animals. Acquisition and use of fetal material has been reviewed by the Stanford University Administrative Panel on Human Subjects in Medical Research and the scope of use does not meet the criteria for research involving human subjects. Anonymized fetal material is provided by the non-profit tissue supply organization Advanced Bioscience Resources, Inc. (ABR) in accordance with applicable federal and state regulations.

### Cells

Human melanoma cells, LDL-GFP melanoma cells, and human embryonic lung fibroblasts (HELFs) were propagated in minimal essential media (MEM) supplemented with 10% fetal bovine serum (FBS) (Invitrogen), nonessential amino acids (100 µM; Omega Scientific), and antibiotics (penicillin, 100 U/mL; streptomycin, 100 µg/mL; Invitrogen) [27]. CHO-K1 Cre cells, which stably express Cre recombinase, were propagated in F-12K Nutrient Mixture with Kaighn's modification (Invitrogen) supplemented with 10% FBS, penicillin, and puromycin (8 µg/mL; Invitrogen) [27].

### Construction of gH(ORF37) expression vectors with mutations in the gHcyt

gH constructs were generated from the pME18s-gH[WT] vector (wildtype gH) or the pME18s-gH[TL] vector, which was a gift from Tadahiro Suenaga and Hisashi Arase (Osaka University, Osaka, Japan) [15], [27]. Primers containing the desired mutation were used to amplify two PCR products using AccuPrime Taq (Invitrogen). The amplicons were digested with either KpnI or AccI restriction enzymes (New England BioLabs), and blunt end ligated with digested pME18s-gH[TL] or the pME18s-gH[WT] vector. Ligated products were electroporated into TOP10F' Electrocomp E. Coli (Invitrogen). Clones were sequenced using the pME18s-KPN1 primer to confirm the mutation. (Table S1: primer list)

### Mutagenesis of gH(ORF37) in pOka-DX bacterial artificial chromosome (BAC) and generation of recombinant viruses

The Y835A, Y835F, TL, Δ834-841, V5, and 834StopV5 mutations were generated in the self-excisable pOka-DX BAC as previously described [27]. Briefly, the mutations were generated in the shuttle vector, pCR669V1-gH-Kan [27]. The modified vector was digested with NaeI and PmeI enzymes (New England BioLabs) and the fragment of interest was inserted into pOka-DX-ΔORF37 BAC, which lacks ORF37 [27], by RED recombination. Fragment insertion was confirmed by PCR using primers {37}F65680-65700 and {37}R68697-68717. The pOka-TK-GFP-gB[Y881F] and pOka-TK-GFP-gH[Δ834-841] BAC constructs were generated from the pOka-TK-GFP-ΔORF37 BAC and -ΔORF31 BAC, respectively, using digested fragments from shuttle vectors following previously described methods [34]. The replacement of TK(ORF36) with TK-EGFP was performed by RED recombination using the previously generated pOka-DX-ΔORF37 and -ΔORF31 BAC and a PCR product amplified with primers 5′-TK-EGFP and 3′-TK-EGFP. The pOka-TK-GFP- gB[Y881F]/gH[Δ834-841] BAC was generated by deleting ORF31 in the pOka-gH[Δ834-841] BAC, replacing the gene with the gB[Y881F] cassette, followed by replacing the ORF36 with the TK-EGFP cassette. To confirm no spurious recombination, vectors were digested with HindIII enzyme (New England BioLabs), separated by gel electrophoresis, and compared to pOka-DX BAC. Recombinant viruses were generated by calcium chloride transfection of melanoma cells with mutant BAC DNA. The mutations in the viral genome were confirmed by PCR and sequencing. Recombinant viruses were passed in HELFs until the MiniF- sequences *Cat* and *SopA* were not detectable by PCR (Table S1), as previously described [35]. PCR of ORF31 further confirmed VZV genomic DNA.

### Immunoprecipitation and western blotting

Briefly, CHO-K1 Cre cells were transfected with gH expressing vectors and the pcDNA3.1-gL vector, using Lipofectamine 2000 (Invitrogen) according to the manufacturer's instructions and lysates were harvested at 24 hours post transfection (hpt) [27]. Lysates from melanoma cells infected with recombinant VZV were harvested at 48 hours post infection (hpi). Immunoprecipitation and western blotting for gH used anti-gH monoclonal antibody SG3 (Meridian Life Science). Western blotting of viral proteins and cellular proteins was performed on infected-melanoma lysates as a control for infection and for sample loading. Membranes were probed for gE (mouse mAb 8612 anti-gE; Millipore), IE63 (rabbit anti-IE63; a kind gift of William Ruyechan, State University of New York, University at Buffalo, Buffalo, NY), and alpha-tubulin (clone B-5-1-2 mouse anti-α-tubulin; Sigma). All primary antibodies were detected using secondary horseradish peroxidase-conjugated antibodies to either anti-mouse or anti-rabbit and ECL Plus Detection Kit (GE Healthcare Bio-Sciences).

### Quantitative Cre reporter assay to measure cell-cell fusion

Cell-cell fusion was measured using the quantitative Cre reporter assay described previously [27], [34]. Briefly, CHO-K1 Cre cells were transfected with equimolar amounts with pCAGGs-gB, a gift from Tadahiro Suenaga and Hisashi Arase (Osaka University, Osaka, Japan) [15], pcDNA3.1-gL, and pME18s-gH mutants and co-cultured with LDL-GFP melanoma cells. The frequency of GFP positive cells indicating fusion events was quantified on a modified Digital FACStar running Diva hardware and software (BD Bioscience). Analysis was performed using FlowJo and all results were normalized to gB/gH[TL]-gL. A negative control that contained only pME18s- and pcDNA3.1-empty vectors was used to establish background levels of GFP expression, which were then subtracted from the fusion frequency data obtained with the test constructs. Experiments were performed at minimum in duplicate.

### Surface expression of gH

Analysis of gH surface expression was performed as previously described [27]. Briefly, CHO-K1 Cre cells were transfected with equimolar amounts of pcDNA3.1-gL and pME18s-gH. Cells were fixed at 24 hours post transfection, stained with anti-gH SG3 antibody, and detected with anti-mouse Alexa Fluor 488 antibody. Analysis was performed on a FACSCalibur controlled by CellQuest Pro (BD Bioscience) and gH expression was quantified with FlowJo (Tree Star). Experiments were performed at minimum in triplicate.

### Confocal microscopy of transfected and infected cells

Confocal microscopy of melanoma cells transfected with pcDNA3.1-gL and either pME18s-gH[WT] or gH mutant constructs was performed as previously described [27]. For infection, melanoma cells were inoculated with 500 plaque forming units (PFU) of recombinant virus. For TK-GFP-BAC transfection, melanoma cells were transfected with pOka-TK-GFP-gB[Y881F], -gH[Δ834-841], and -gB[Y881F]/gH[Δ834-841] BACs using Lipofectamine 2000. Cells were fixed with 4% paraformaldehyde at post 24 hours for non-BAC transfected and infected cells and 72 hours for BAC transfected cells. Cells were probed for gH, early endosomes and the trans-Golgi-network using anti-gH mAb SG3 (mouse), anti-EEA1 mAb (rabbit; Novus Biological), and anti-TGN46 pAb (sheep; AbD Serotec), respectively. TK-GFP-BAC-transfected cells were probed with anti-IE62 (mouse; Chemicon International) and anti-ORF23 (polyclonal rabbit; [36]). Primary antibodies were detected with secondary antibodies, anti-mouse Alexa Fluor 555 (Invitrogen), anti-rabbit Alexa Fluor 488 (Invitrogen), and anti-sheep Alexa Fluor 647 (Invitrogen). Nuclei were stained with Hoechst 33342 (Invitrogen). Images were captured with a Leica SP2 AOBS Confocal Laser Scanning Microscope. Channel merging and image processing was performed with ImageJ and Photoshop.

### Brightfield and fluorescence microscopy of syncytium

Melanoma cells were seeded in 6-well plates and infected with 500 PFU of pOka or recombinant gH mutant viruses. Images of syncytium were taken at 24, 36, and 48 hours post infection. Prior to each time point, infected cells were incubated with fresh media supplemented with Hoechst 33342 at 1∶1000 dilution for 10 minutes. Brightfield and fluorescence microscopy images were taken with an AX10 Microscope (Zeiss) equipped with an X-Cite Series 120 fluorescence excitation light source (Lumen Dynamics). Images were aligned and processed in Photoshop to enhance contrast for the cytoplasmic regions of the syncytium. To quantify the number of nuclei per syncytium at 36 hours post infection (hpi), 15 syncytia were randomly selected and the nuclei were visually counted in a single plane from images taken by a light microscope.

### Replication kinetics, virus titration, and plaque size

As previously described [37], melanoma cells were infected with 1000 PFU of recombinant VZV and harvested at 24 hour intervals to examine replication kinetics. Viral titration was performed by 10-fold dilution on melanoma cells in triplicate. To access plaque size, stained plaques (n = 30) from titration plates at four days post infection were captured with an AX10 Microscope (Zeiss) with the plaques outlined and area calculated using ImageJ, as described [34].

### Transmission electron microscopy of VZV-infected or VZV BAC-transfected melanoma cells

To examine infected cells, 12 mm glass coverslips were seeded with 2×10^5^ melanoma cells and infected with 1000 PFU of pOka-gH[Δ834-841] or pOka virus. To examine cells transfected with pOka-TK-GFP-gB[Y881F], -gH[Δ834-841], and -gB[Y881F]/gH[Δ834-841] BACs, 100 mm dishes seeded with 1.2×10^6^ melanoma cells were transfected with 30 µg of BAC DNA using Lipofectamine 2000. At 24 hours post transfection, cells were harvested and sorted for GFP positive cells using a BD InFlux Special Order sorter for enrichment. Sorted cells were then added to a cloning ring placed upon a 12 mm glass coverslip preseeded with 4×10^5^ melanoma cells. At 48 hours post reseeding for transfected cells and 72 hpi for infected cells, samples were fixed with 2% glutaraldehyde and 4% p-formaldehyde in 0.1M sodium cacodylate buffer pH 7.2 for 20 minutes. Cells were then treated with 1% osmium (OsO_4_) for one hour and stained with 1% uranyl acetate overnight. Cells were dehydrated with multiple incubations of increasing concentrations of ethanol (50%, 70%, 95%, and 100%) followed by acetonitrile. Epon infiltration was performed by subsequent incubations with 1∶1 (Epon/Acetonitrile), 2∶1 (Epon/Acetonitrile), and 100% Epon. Samples were incubated at 65°C to allow for polymerization. Glass coverslips were dissolved by incubation in 49% hydrofluoric acid for 20 minutes. Areas of interest were trimmed. Ultrathin sections were prepared using an ultratome (Leica Microsystems) and placed upon formvar carbon film 100Mesh copper grids (Electron Microscopy Sciences). Grids were stained with 4% uranyl acetate and 0.2% lead citrate. Sections were visualized using a JEOL 1400 transmission electron microscope at 80 kV and digital photographs were captured with a Gatan Multiscan 701 digital camera.

### Evaluation of gH mutant viruses in human skin xenografts

Skin xenografts were prepared in homozygous CB-17 scid/scid mice using human fetal skin tissue obtained according to federal and state regulations as previously described [12]. Briefly, implanted skin xenografts were inoculated with HELFs infected with wildtype pOka or mutant viruses. The inoculum titer for each virus was determined to confirm similar PFU/mL concentrations. Xenografts were harvested at 10 and 21 days post infection (dpi) and viral titers were determined. Confocal microscopy of sectioned infected skin xenografts was performed as previously described [34]. Briefly, 5 µm sections were deparaffinized and treated with citrate-based-Antigen Unmasking Solution (Vector) at high temperature following the manufacturer's instructions. Sections were then probed with primary antibodies, anti-gE Mab8612 (mouse; EMD Millipore), anti-ORF23 (polyclonal rabbit; [36]), and anti-TGN46 (sheep; AbD Serotec). Primary antibodies were detected with anti-mouse Alexa Fluor 555, anti-rabbit Alexa Fluor 488, and anti-sheep Alexa Fluor 647. Nuclei were stained with Hoechst 33342. Images were aligned and processed with Photoshop.

### Transmembrane and cytoplasmic domain predictions

Amino acid sequences of gH homologues were obtained from protein searches on PubMed. Sequences were submitted to TOPCONS (http://topcons.cbr.su.se/) to identify transmembrane and cytoplasmic domains [38]. Hydrophobic residues in the predicted cytoplasmic domains were manually identified.

### Statistical Analysis

All quantitative results were analyzed by either one-way or two-way ANOVA to determine statistical significance using Prism (Graphpad Software).

## Results

### Amino acids 834-841 of the VZV gH cytoplasmic domain are critical for the regulation of cell fusion mediated by the gB/gH-gL complex

**Figure 1:**
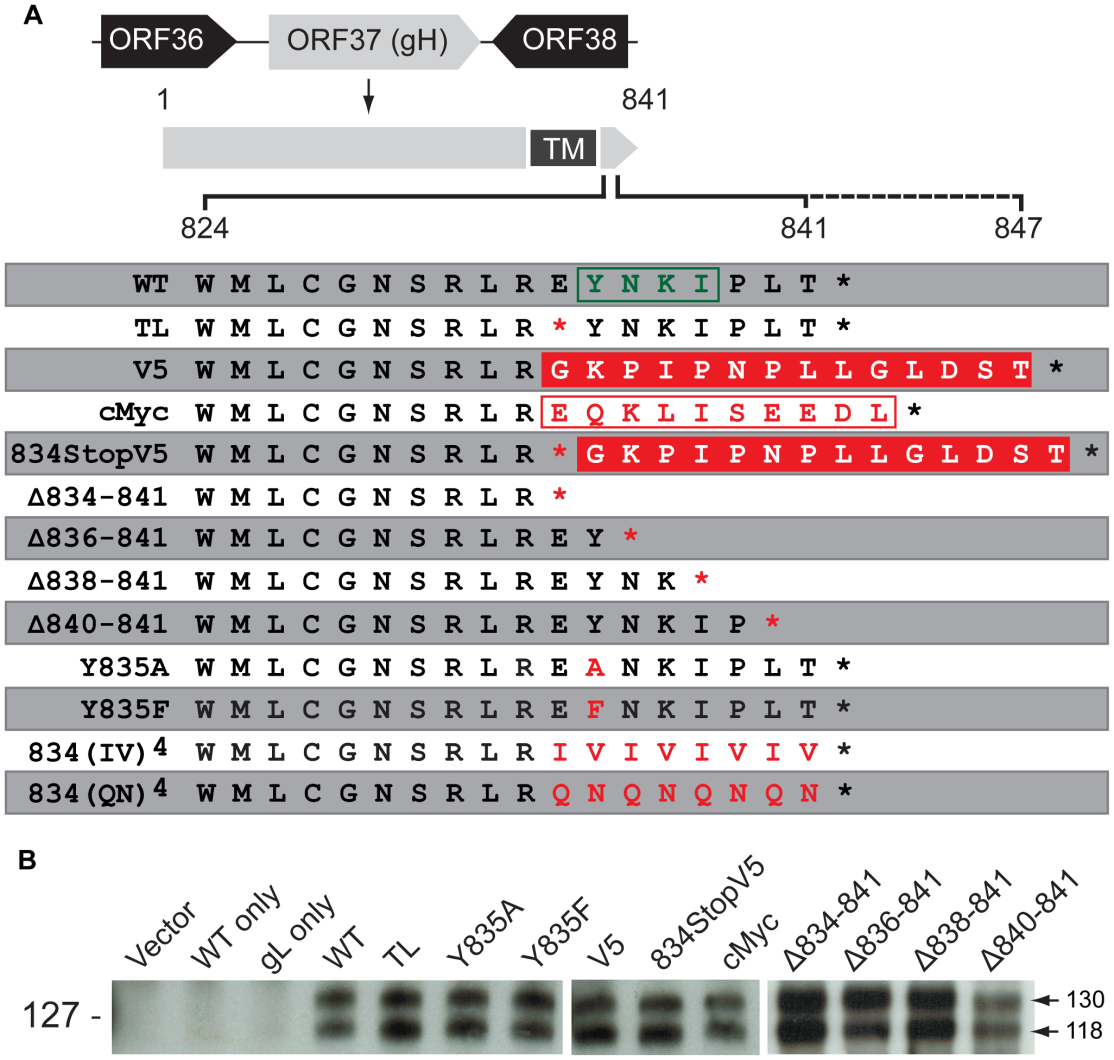
Construction and *in vitro* expression of VZV gH mutants. (A) VZV gH constructs with mutations in the predicted cytoplasmic domain (824-841) [29]. The solid black line indicates the length of the wildtype (WT) gHcyt (824-841) while the dotted line represents extension of the gHcyt length in the V5 and cMyc constructs. The predicted endocytosis YXXΦ motif is boxed in green. The V5 and cMyc sequences are boxed in solid red and white, respectively. The red characters indicate mutations made to the gHcyt. Asterisks represent stop codons. TM indicates the predicted transmembrane domain. (B) Western blot of gH from immunoprecipitation using anti-gH(SG3) antibody of lysates from CHO-K1 Cre cells transiently expressing vector only (Vector), wildtype gH only (WT only), gL only (gL only) or gH mutants with gL. Arrows indicate two molecular weights of gH, 118 and 130 kilodaltons (kDa). Molecular mass standard is indicated on the left.

**Figure 2:**
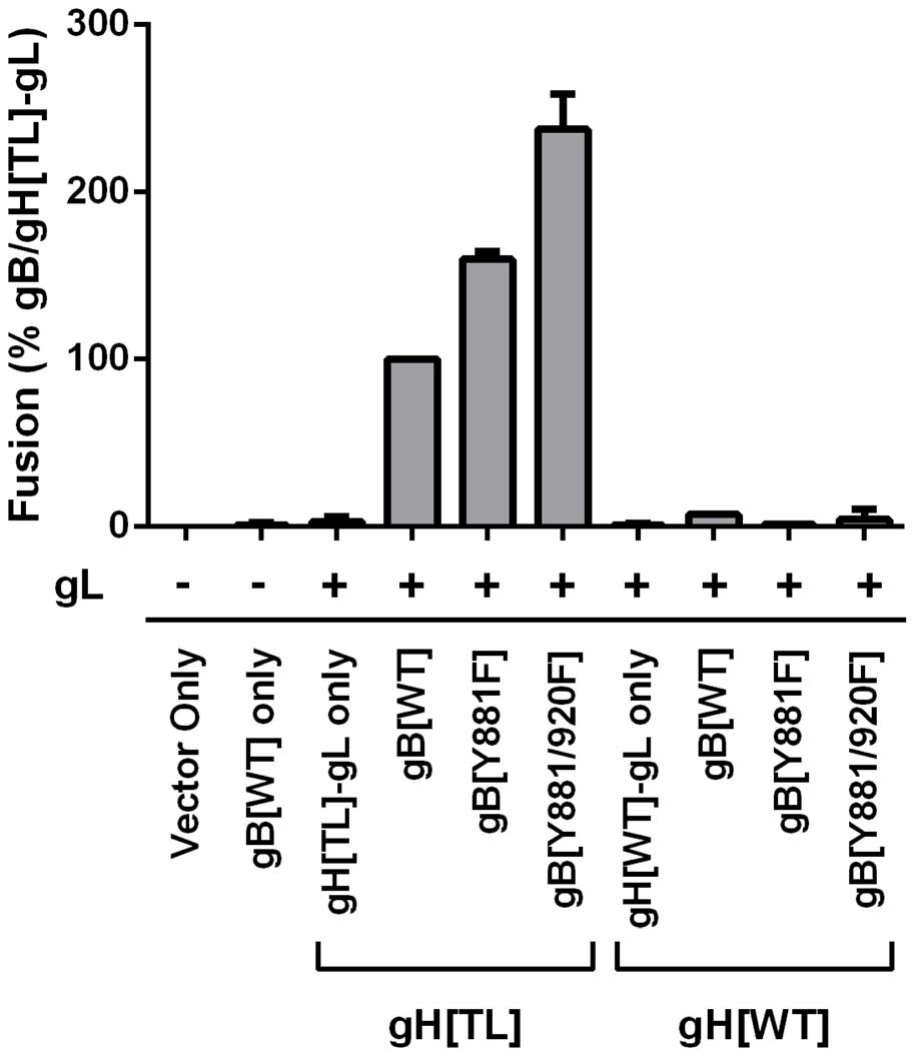
Truncation of the gHcyt causes enhanced *in vitro* cell-cell fusion. Cell-cell fusion induced by gB and gH mutants were quantified by the Cre reporter fusion assay, with fusion events represented as a percentage relative to fusion events of gB/gH[TL]-gL. gB[WT], gB[Y881F], and gB[Y881/920F] were coexpressed with either gH[TL] or gH[WT] and gL. Vector only represents transfection with empty vectors. gB[WT] only represents expression of gB[WT] with empty vectors. gH[TL]-gL only and gH[WT]-gL only represent expression of gH[TL] and gH[WT] with gL, respectively, with an empty vector. The plus and minus symbols indicate the presence or absence of gL expressing vector, respectively. Data shown represents the mean of two independent experiments with the whiskers indicating the standard error of the mean (SEM). All values were evaluated by ANOVA analysis and found to be significantly different from gB/gH[TL]-gL (P<0.001).

Expression of VZV gB, gL, and gH with amino acids 834-841 of its cytoplasmic domain absent (gH[TL]) because of a E834Stop mutation has been demonstrated to produce a high frequency of cell fusion events by the quantitative Cre reporter assay [27], [34]. This assay allows for the identification of domains that are required for fusion regulation based on enhanced fusogenicity (hyperfusogenicity) conferred by the mutations [34]. In contrast to gH[TL], the fusion frequencies for wildtype gH (gH[WT]) were only marginally greater than the vector alone indicating that the full length gH protein limits fusion under these conditions (Figure 1A and 2). Co-expression of gH[TL] with only gL did not induce fusion, further confirming that gB was a critical component of the cell fusion machinery. Expression levels and the molecular weights of gH[TL] were similar to gH[WT] in CHO cells, based on immunoprecipitation and western blots (Figure 1B), demonstrating that amino acids 834-841 in the gHcyt do not affect the synthesis, stability, or the post-translational processing of gH into its 118 or 130 kDa mature form in the transfected CHO cells used to evaluate cell fusion.

To determine whether the gHcyt was critical for cell fusion regulation in combination with the gBcyt, the fusogenicity of the single substitution, gB[Y881F], and double substitution, gB[Y881/920F], were examined with gH[TL] and gH[WT] (Figure 2). When co-expressed with gH[TL] and gL, the gB[Y881F] and gB[Y881/920F] mutants induced levels of fusion at 60% and 137% greater than wildtype gB (gB[WT]), respectively, which was consistent with our previous report [34]. In contrast, both gBcyt mutants had fusion levels that were limited or below the level of detection when they were co-expressed with gH[WT]. Thus, the hyperfusion phenotype of gB[Y881F] and gB[Y881/920F] is dependent upon the absence of amino acids 834-841 of gH, suggesting that the cytoplasmic domains of gB and gH might function together in regulating cell fusion.

### Regulation of gB/gH-gL mediated cell fusion by the gHcyt is dependent on the physical length of the domain

**Figure 3:**
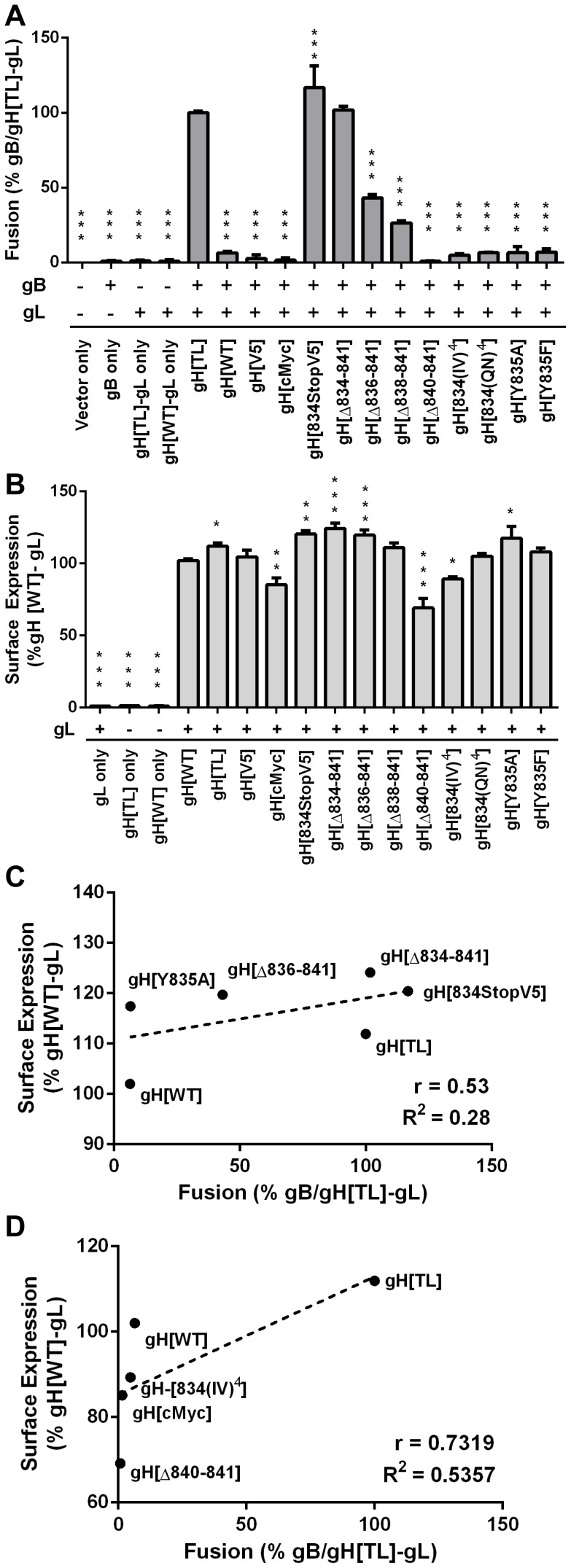
Regulation of cell-cell fusion by gH is dependent on cytoplasmic domain length but motif independent. (A) Cell-cell fusion between melanoma cells and CHO-K1 Cre cells expressing either gH[WT], gH[TL], gH[V5], gH[cMyc], gH[834stopV5], gH[Δ834-841], gH[Δ836-841], gH[Δ838-841], gH[Δ840-841], gH[834(IV)^4^], gH[834(QN)^4^], gH[Y835A], or gH[Y835F] with wildtype gB and gL. Fusion was quantified by the Cre reporter assay with fusion events represented as a percentage relative to fusion events of gB/gH[TL]-gL. Vector only represents transfection with empty vectors. gB only represents expression of gB with empty vectors. gH[TL]-gL only and gH[WT]-gL only represent expression of gH[TL] and gH[WT] with gL, respectively, with an empty vector. The plus and minus symbols indicate the presence or absence of gB or gL expressing vectors, respectively. (B) gH cell surface expression of melanoma cells transiently expressing gH mutants with gL quantified by nonpermeabilized staining for gH and flow cytometry. Surface expression is represented as a percentage relative to gH[WT]-gL. gL only represents expression of gL with empty vector. gH[TL] only and gH[WT] only represent expression of the two vectors with an empty vector. The plus and minus symbols indicate the presence or absence of gL expressing vectors, respectively. Data shown represents the mean of three independent experiments with the error bars indicating the SEM. Statistical differences between the mutants and gB/gH[TL]-gL for the cell-cell fusion assay and gH[WT]-gL for surface expression were evaluated by ANOVA (*P<0.05, **P<0.01, ***P<0.001). gH mutants with mean surface expression statistically (C) higher or (D) lower than gH[WL]-gL were examined on scatter plots comparing their mean surface expression relative to gH[WT]-gL and mean fusion levels relative to gB/gH[TL]-gL. The dotted line represents the trend line with Pearson r and R^2^ values noted for each graph.

To further characterize regulation of cell fusion by the gHcyt, mutants were constructed that replaced the eight amino acids of 834-841 with either the 14 amino acid V5 epitope (gH[V5]) or the 10 amino acid cMyc epitope (gH[cMyc]) (Figure 1A). To confirm that there was no read through of the E834Stop nonsense mutation or disruption of the expression plasmid by the V5 sequence, a mutant that contained both the E834Stop and V5 epitope (gH[834StopV5]) was also constructed. All three gH mutants had protein expression profiles similar to gH[WT] in transfected CHO cells (Figure 1B). However, despite the absence of amino acids 834-841, the V5 and cMyc mutations resembled gH[WT] in having very limited capacity to induce cell fusion (Figure 3A). Similar to the increase in fusion levels observed by gH[TL] relative to gH[WT], gH[834StopV5] also had increased fusion in comparison to its corresponding control, the gH[V5]. Although a slight increase in fusion levels was observed between gH[834StopV5] and gH[TL], possibly because of the V5 sequence, the enhanced fusion levels caused by the gH[834StopV5] mutation demonstrated that read through past the 834Stop codon, like the gH[TL] mutation, did not occur. This strongly suggests that the gHcyt length is important for regulating cell fusion.

To further determine the importance of the physical length of the gHcyt rather than its sequence for regulating cell fusion, a series of truncation mutants was constructed: gH[Δ834-841], gH[Δ836-841], gH[Δ838-841], and gH[Δ840-841], containing eight, six, four, and two amino acid deletions from the carboxyl terminus of the gHcyt, respectively (Figure 1A). None of the truncation mutations changed the protein expression profile of gH in CHO cells (Figure 1B). The incremental deletions of the gHcyt caused stepwise increases in gB/gH-gL mediated cell fusion rather than an all-or-none increase in fusion levels that would be indicative of deletion of a specific functional motif (Figure 3A). As expected, the gH[Δ834-841] mutant induced levels of cell fusion similar to gH[TL], confirming again the importance of amino acids 834-841 for fusion regulation. This also demonstrated that the induction of cell fusion by gH[TL] was not caused by the type of mutation, whether nonsense or deletion, that was employed to eliminate expression of amino acids 834-841. The remaining Δ836-841, Δ838-841 and Δ840-841 deletions exhibited 56%, 73%, and 99% less cell fusion when compared with gH[TL], respectively (Figure 3A). Thus, this data provided further evidence that regulation of gB/gH-gL mediated cell-cell fusion by the gHcyt depends on the length of the cytoplasmic domain and not the specific sequence or intrinsic properties of the amino acids in the domain.

The presence of hydrophobic residues in the cytoplasmic domain of viral fusion-related proteins has been suggested to have a role in regulating cell fusion [39]. The predicted gHcyt of alphaherpesvirus homologues, including VZV, contain highly hydrophobic residues (leucine, isoleucine, and, phenylalanine) (Figure S1), suggesting that the presence of hydrophobic residues in the gHcyt might be a regulatory factor for cell fusion. To test this hypothesis, two gH mutants were constructed, gH[834(IV)^4^] and gH[834(QN)^4^], in which amino acids 834-841 were substituted with a series of alternating isoleucine and valine residues or glutamine and arginine residues, respectively (Figure 1A). The 834(IV)^4^ substitution was highly hydrophobic, while the 834(QN)^4^ was highly hydrophilic (Figure S2A). Similar to gH[WT], both mutations induced little or no detectable fusion (Figure 3A), which demonstrated that fusion regulation by the gHcyt was not dependent upon the presence of hydrophobic residues and further confirmed that the regulation was motif independent.

Given that expression of gH on the surface of the cell would be expected to influence cell fusion, FACS analysis with anti-gH antibody was performed on CHO cells expressing the gH[WT] or gH mutants along with gL (Figure S3) (Figure 3B). gH[TL] and gH[WT] protein were not detected on the cell surface in the absence of gL, which was consistent with previous findings [27]. Surface expression levels of the gH mutants with gL could be separated into three distinct groups, with mutants having significantly greater, equal, or reduced surface expression compared to gH[WT]. gH[TL], gH[834StopV5], gH[Δ834-841], and gH[Δ836-841] exhibited surface expression levels that were 12%, 20%, 24%, and 19% greater than gH[WT], respectively. gH[V5] and gH[834(QN)^4^] mutants had surface expression levels equivalent to gH[WT], while gH[cMyc], gH[Δ840-841], and gH[834(IV)^4^] had surface expression levels that were 15%, 30%, and 10% less than gH[WT] (Figure 3B). To determine if there was a correlation between the surface expression and cell fusion effects of these mutants, the mean levels of their surface expression were plotted against their cell fusion levels on a scatter plot along with gH[WT] and gH[TL]. For both the greater and lesser surface expression groups, no significant correlation was found between their surface expression and cell fusion levels. A Pearson R value of 0.53 and R^2^ value of 0.28 was determined for the mutants with increased surface expression relative to gH[WT] with a P value of 0.29 (Figure 3C) and while a high R value of 0.713 and R^2^ value of 0.54 was calculated for those mutants with reduced surface expression, the P value was 0.15, making the correlation not statistically significant (Figure 3D). Thus, while gB-gH/gL mediated cell fusion requires the presence of gH on the surface of the cell, greater levels of surface expression did not correlate with the induction of cell fusion and the induction or extent of fusion was not a result of increased gH surface expression.

### Loss of cell fusion regulation is not a result of aberrant gH intracellular trafficking

**Figure 4:**
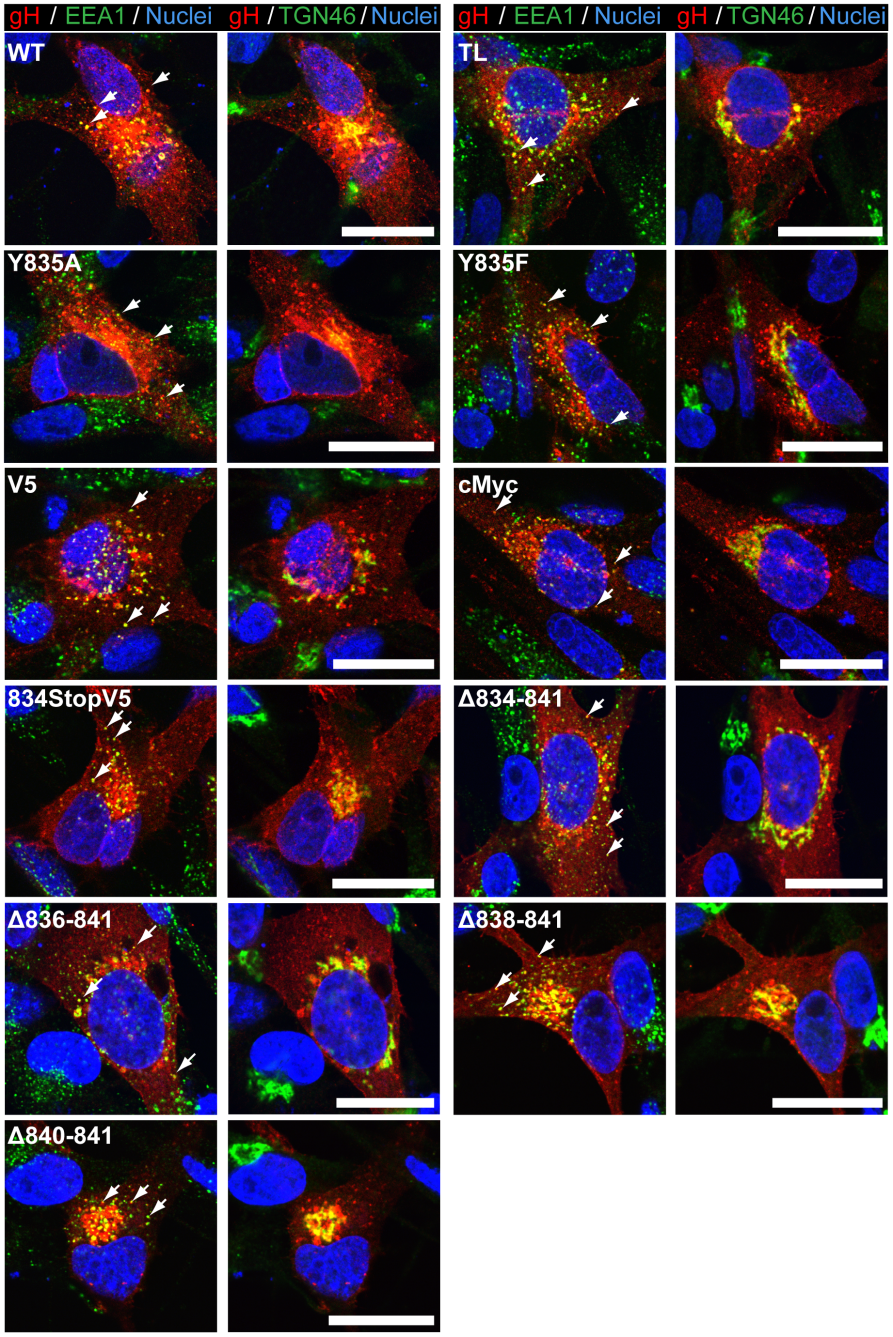
Amino acids 834-841 of the gHcyt are dispensable for intracellular trafficking and endocytosis of gH *in vitro*. Confocal microscopy of melanoma cells transiently expressing gH[WT] or gH mutants with gL at 24 hours post transfection. Cells were stained for gH (red), early endosome antigen (EEA1; green), trans-Golgi network (TGN46; green), and nuclei (Hoechst 33342; blue). White arrows indicate colocalization of EEA1 and gH, which highlight representative endocytic vesicles containing gH. The scale bars represent 20 µm.

To determine if the changes in cell fusion associated with the gHcyt mutations might be due to altered intracellular trafficking, the subcellular localization of gH mutants in melanoma cells co-expressing gL was examined by confocal microscopy. As expected from previous reports, gH[WT] had a pattern of juxta-nuclear localization as well as a punctate localization in the cytoplasm [27] (Figure 4). Co-staining with organelle markers confirmed the co-localization of gH with the trans-Golgi network (anti-TGN-46) at the juxta-nuclear position and with some early endosomes, detected with anti-EEA1 antibody, appearing as punctae in the cytoplasm. gH[WT]-like subcellular localization was also observed for the gH[V5], gH[cMyc], gH[834StopV5], gH[Δ834-841], gH[Δ836-841, gH[Δ838-841], and gH[Δ840-841] mutants (Figure 4) and for gH[834(IV)^4^] and gH[834(QN)^4^] (Figure S2B). Thus, amino acids 834-841 of the gHcyt did not have a functional role in gH intracellular trafficking or its internalization from the cell surface.

### The gHcyt YXXΦ motif does not regulate cell fusion

The role of the ^835^YNKI^838^ motif was examined in the virus-free transfection system by constructing two mutants with the tyrosine (Y835) residue substituted with either alanine (Y835A) or phenylalanine (Y835F). The alanine substitution fully disrupted the residue, while the phenylalanine substitution served to determine if the hydroxyl group had a functional role in endocytosis (Figure 1A). Neither substitution affected levels of gH protein expression or processing compared to gH[WT] (Figure 1B). Similar to gH[WT], both gH[Y835A] and gH[Y835F] were able to colocalize with EEA-1 markers, indicating that the protein was present in endocytic vesicles (Figure 4, white arrows). Thus, gH endocytosis was not prevented by disrupting the YXXΦ motif.

Similar to gH[WT], both the Y835A and Y835F substitutions induced little to no detectable fusion in the Cre reporter fusion assay (Figure 3A). Cell surface expression levels were different between the mutants, with gH[Y835A] having levels of surface expression greater than gH[WT] and gH[Y835F] having levels similar to the gH[WT] (Figure 3B). This further confirmed the lack of correlation between the induction of cell fusion observed in the Cre reporter fusion assay and increased gH surface expression. These results demonstrated the ^835^YNKI^838^ endocytosis motif in the gHcyt did not contribute to regulation of gB/gH-gL mediated cell fusion in the absence of other VZV proteins and was consistent with evidence that the regulation of gB/gH-gL mediated cell fusion depended on the physical length of the gHcyt and not the presence of specific residues or motifs.

## Truncation of the gHcyt enhances VZV syncytia formation while reducing viral titers

**Figure 5:**
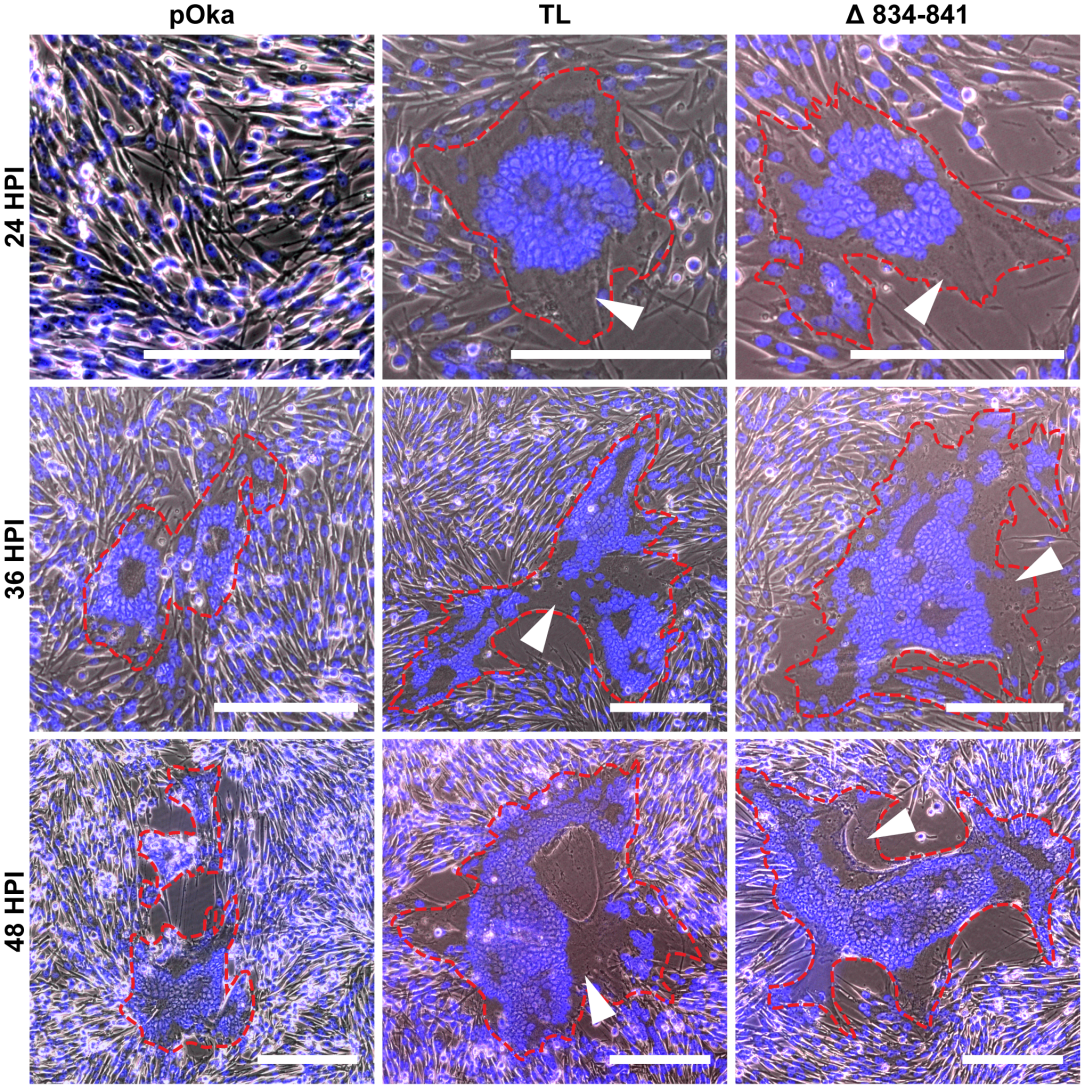
Truncation of the gHcyt causes exaggerated syncytia formation in VZV-infected melanoma cells. Merged phase contrast and fluorescence microscopy images of melanoma cells infected with pOka, pOka-gH[TL], and pOka-gH[Δ834-841] viruses at 24, 36, and 48 hours post infection. Nuclei were stained with Hoechst 33342 (blue). Visually detectable syncytia are outlined in red with white arrows indicating extended cytoplasm of VZV-induced syncytia. The scale bars represent 200 µm.

To determine the role of the gHcyt in VZV-induced syncytia formation and replication kinetics, six recombinant viruses were generated by inserting the mutations, Y835A, Y835F, V5, 834StopV5, Δ834-841, and TL into a parental Oka (pOka) BAC [28]. Infectious virus was recovered from all of the BACs with mutant gH. To determine if amino acids 834-841 of gH were critical for syncytia formation regulation, syncytium morphology was examined in VZV infected-melanoma cells at 24, 36, and 48 hours post infection (hpi). Mutant viruses expressing gH without amino acids 834-841, including pOka-gH[TL] and pOka-gH[Δ834-841], produced syncytia with a larger number of nuclei as early as 24 hpi, while few to no syncytia were observed at the same time point in cells infected with pOka (Figure 5). At 36 hpi, pOka-gH[TL], pOka-gH[Δ834-841], and pOka all exhibited syncytia formation, but the morphology of the pOka-gH[TL] and pOka-gH[Δ834-841] syncytia was significantly different compared to pOka. Melanoma cells infected with either of the gH mutants exhibited not only an observable increase in the number of nuclei per syncytium, but also had extended cytoplasm in the syncytia (Figure 5, white arrows). When quantifying the number of nuclei at 36 hpi, pOka-gH[Δ834-841] syncytium had 122±38.5 nuclei compared to 40±16.8 nuclei for pOka syncytium (Figure S4). At 48 hpi, there was a further increase in number of nuclei and enlargement of the extended cytoplasm within each syncytium. Syncytia formation and morphology of pOka-gH[834stopV5] was similar to the viruses with the TL and Δ834-841 mutations (data not shown). The syncytia morphology in melanoma cells infected with pOka-gH[V5], pOka-gH[Y835A], and pOka-gH[Y835F] viruses was similar to pOka at 36 and 48 hpi, indicating that these mutations had no effect on syncytia formation or morphology (Figure S5). The number of nuclei in pOka-gH[Y835A] induced syncytium at 36 hpi was 36±13.8, which was comparable to pOka (Figure S4). Thus, the truncation of the gHcyt caused an exaggerated syncytia formation phenotype in VZV infected melanoma cells, correlating with the increase in fusion levels observed in the Cre reporter fusion assay.

**Figure 6:**
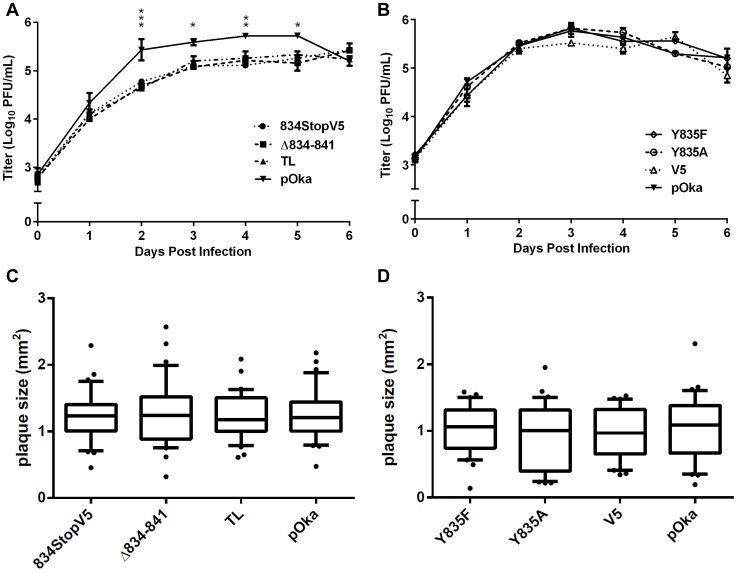
Truncation of the gHcyt reduces viral titers in melanoma cells. (A) Replication kinetics of pOka-gH[834StopV5], pOka-gH[Δ834-841], pOka-gH[TL], and pOka in melanoma cells over six days. Individual points represent the mean of harvested infected cells titrated in triplicate, including the titrated inoculum at Day 0. Titers were measured in log_10_ of plaque forming units per milliliter (PFU/mL). The error bars represent the SEM. Statistical differences between pOka-gH[TL] and the other viruses were evaluated by two-way ANOVA (*P<0.05, **P<0.01, ***P<0.001). (B) Replication kinetics of pOka-gH[Y835A], pOka-gH[Y835F], pOka-gH[V5], and pOka in melanoma cells over six days with no statistical differences were observed between pOka and the mutant viruses. (C and D) Box and whisker plots of plaque sizes measured (n = 30) in melanoma cells infected with (C) pOka-gH[834StopV5], pOka-gH[Δ834-841], pOka-gH[TL], and pOka or (D) pOka-gH[Y835A], pOka-gH[Y835F], pOka-gH[V5], and pOka at four days post infection. Area of plaques was measured in millimeter squared (mm^2^). The boxes represent the 10–90% percentile with the median indicated by the band and the outliers (<10% or >90% percentile) represented as individual black dots.

To determine if the gHcyt truncation inhibited propagation of the virus, replication kinetics of the gHcyt mutant viruses and pOka were compared in melanoma cells. Viruses with deletions that induced high levels of fusion in the Cre reporter fusion assay (834StopV5, Δ834-841, and TL) and exaggerated syncytia formation had a statistically significant 0.5–1 log_10_ decrease in viral titers between two and five days post infection (dpi) (Figure 6A), indicating that amino acids 834-841 of the gHcyt were important for viral propagation. In contrast, titers of viruses with mutations in the gHcyt that failed to induce fusion or altered syncytia formation *in vitro* (Y835A, Y835F, and V5) were similar to pOka (Figure 6B). The decrease in titer of pOka observed at six dpi was a result of a >95% infection of the monolayer. Thus, effective viral propagation is dependent upon canonical regulation of syncytia formation by the gHcyt.

**Figure 7:**
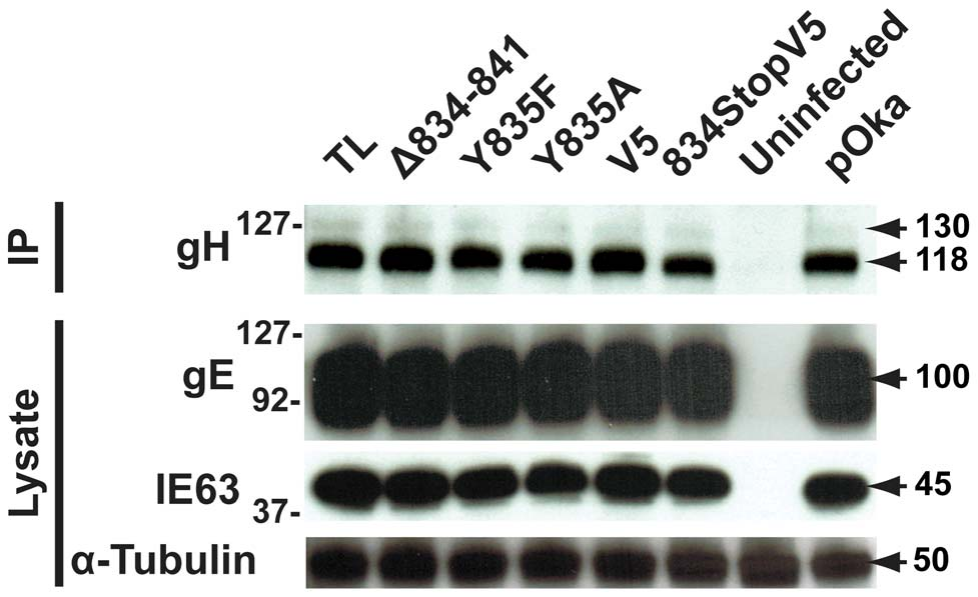
Amino acids 834-841 of the gHcyt are dispensable for gH maturation during infection of melanoma cells. Western blot of gH immunoprecipitated (IP) from lysates of melanoma cells either mock-infected (uninfected) or infected with pOka-gH[Δ834-841], pOka-gH[TL], pOka-gH[Y835A], pOka-gH[Y835F], pOka-gH[V5], pOka-gH[834StopV5], or pOka for 48 hours. To determine the level of expression of essential viral proteins, the lysates from the same infected cells were also probed for IE63 and glycoprotein E (gE). Alpha tubulin was probed as a loading control. Arrows indicate molecular weights of gH, gE, IE63, and α-tubulin. Molecular weight standard is indicated on the left. All molecular weights are in kilodaltons (kDa).

**Figure 8:**
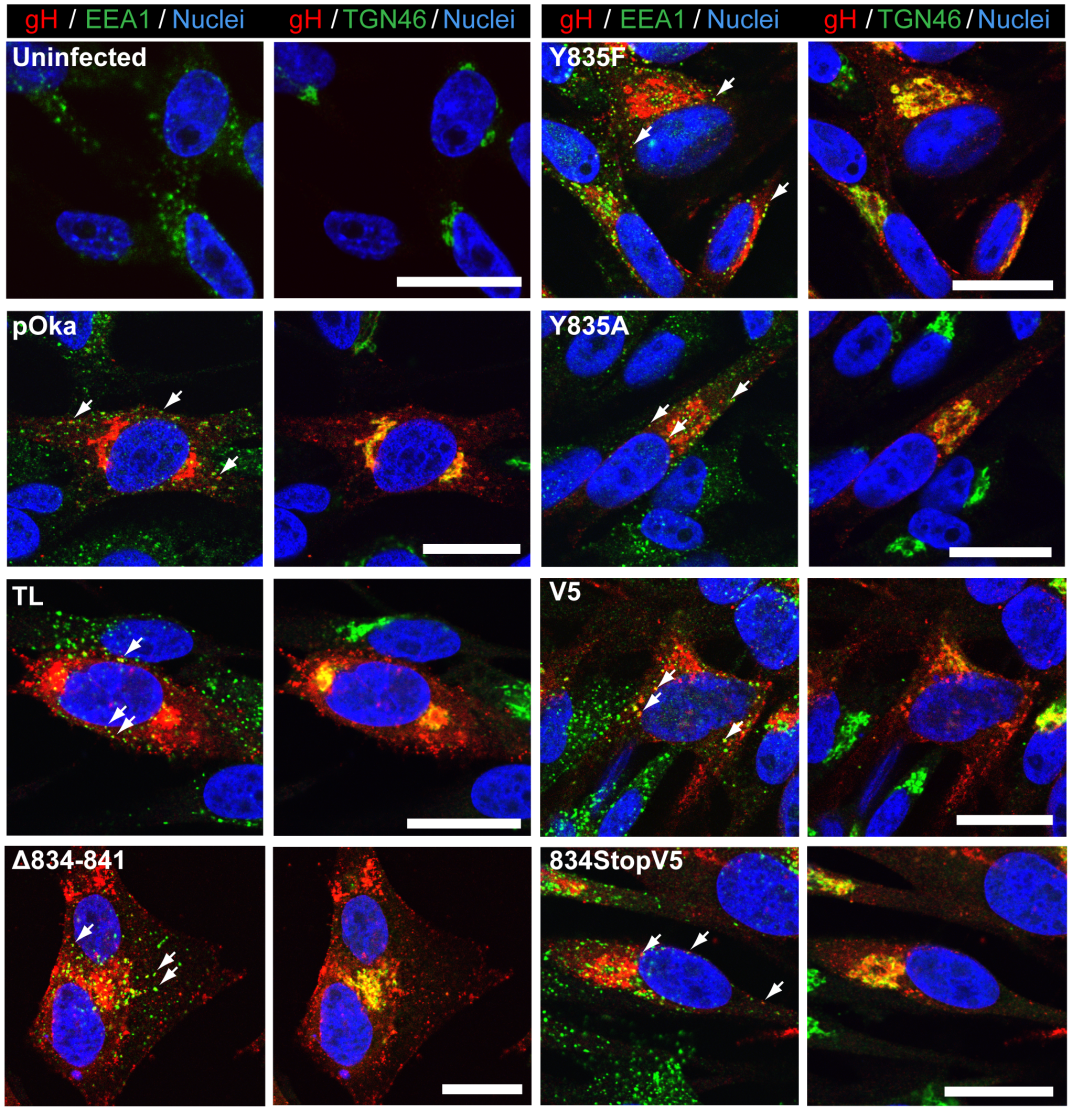
Amino acids 834-841 of the gHcyt are dispensable for intracellular trafficking and endocytosis of gH during infection of melanoma cells. Confocal microscopy of melanoma cells at 24(Uninfected) or infection with pOka-gH[Δ834-841], pOka-gH[TL], pOka-gH[Y835A], pOka-gH[Y835F], pOka-gH[V5], pOka-gH[834StopV5], or pOka viruses. Cells were stained for gH (red), early endosome antigen (EEA1; green), trans-Golgi network (TGN46; green) and nuclei (Hoechst 33342; blue). White arrows indicate colocalization of EEA1 and gH, which highlight representative endocytic vesicles containing gH. The scale bars represent 20 µm.

To elucidate the cause of the reduction in infectious virus production, the plaque size, viral protein expression, and gH intracellular trafficking of the gHcyt mutant viruses were examined. The plaque sizes for pOka-gH[834StopV5], pOka-gH[Δ834-841], and pOka-gH[TL] viruses were similar to pOka and the other mutants (Y835A, Y835F, and V5) at four dpi (Figure 6C and D), indicating that deletion or substitution of amino acids 834-841 of the gHcyt did not inhibit viral spread to its neighboring cells *in vitro* but caused a premature fusion of infected cells. The maturation of gH during infection was not inhibited by either deletions or substitutions of the gHcyt as demonstrated by detection of both the 118 and 130 kDa molecular weight forms like pOka (Figure 7) indicating that the dysregulation of syncytia formation was not related to altered gH maturation. In contrast to transfected CHO cells, the levels of expression of the 130 kDa molecular weight form of gH[WT] and gH mutants during infection of melanoma cells was less than the 118 kDa form (Figure 1B). This difference in expression of the 130 kDa form did not noticeably affect fusion, since the exaggerated syncytia formation phenotype of pOka-gH[834StopV5], pOka-gH[Δ834-841], and pOka-gH[TL] was consistent with the data of the Cre reporter assay. Differences in glycosylation in mammalian cells lines [40] or posttranslational modification during infection that could affect gH processing or stability of the 130 kDa form did not alter the fusion phenotype. Intracellular trafficking of gH during infection of melanoma cells was not affected by the gHcyt substitutions when examined by confocal microscopy (Figure 8). Consistent with the localization of transiently expressed gH[WT] and gL, gH mutant proteins colocalized with markers for the TGN (anti-TGN46) and a subset of the early endosomes (anti-EEA1), indicating that gH was endocytosed during infection. Expression of VZV immediate early and early proteins were unaffected by the gHcyt mutations as demonstrated by pOka-like expression levels of immediate early 63 (IE63) and glycoprotein E, an early gene product, in western blots from infected cell lysates (Figure 7). Because changes were not detected in viral spread, gH protein expression and trafficking or synthesis of essential viral proteins, the reduction in infectious virus production by pOka-gH [TL], -gH[Δ834-841], and -gH[834StopV5] mutants was attributed to the exaggerated syncytia formation.

## Viral particle formation and egress are unaffected by the truncation of the gHcyt

**Figure 9:**
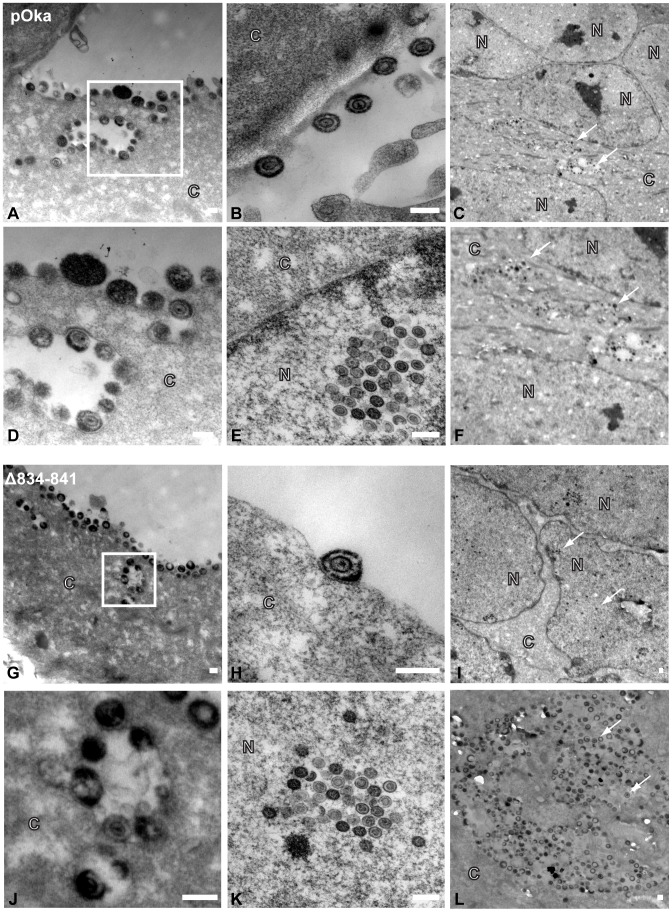
Truncation of the gHcyt does not affect VZV particle formation or egress. Electronmicrographs of melanoma cells infected with (A–F) pOka or (G–L) pOka-gH[Δ834-841] at 48 hours post infection. (A) (G) Images of viral particles on the cell surface, with micrographs D and J represent magnified images of the boxed area of micrographs A and G, respectively. Representative images of (B) (H) viral particle, (E) (K) crystalline arrays of nucleocapsids, (C) (I) nuclei, and (F) (L) vesicles containing viral particles. The C and N denote cytoplasmic and nuclear space, respectively, with the white arrows indicating representative capsids. The scale bar represents 200 nm.

To determine if viral particle formation or egress was affected by the deletion of amino acids 834-841, electron micrographs were taken of pOka and pOka-gH[Δ834-841] infected melanoma cells at 48 hpi. Typical virus particles were observed on the surface of pOka-gH[Δ834-841]-infected cells (Figure 9G and J) and found to be similar to those observed on pOka-infected cells (Figure 9A and D) demonstrating that the deletion did not inhibit egress of virus particles. The pOka and pOka-gH[Δ834-841] viral particles had similar size and morphology (Figure 9B and H) indicating that the deletion did not affect particle formation. Nucleocapsids of pOka-gH[Δ834-841] formed crystalline arrays (Figure 9E) like pOka (Figure 9K) and no accumulation of nucleocapsids were observed in the perinuclear space, indicating that the gHcyt deletion did not inhibit nuclear egress (Figure 9C and I). pOka and pOka-gH[Δ834-841] particles were also found in cytoplasmic vesicles (Figure 9F and L).

## Truncation of the gHcyt in conjunction with disrupting the gBcyt ITIM significantly limits viral spread

**Figure 10:**
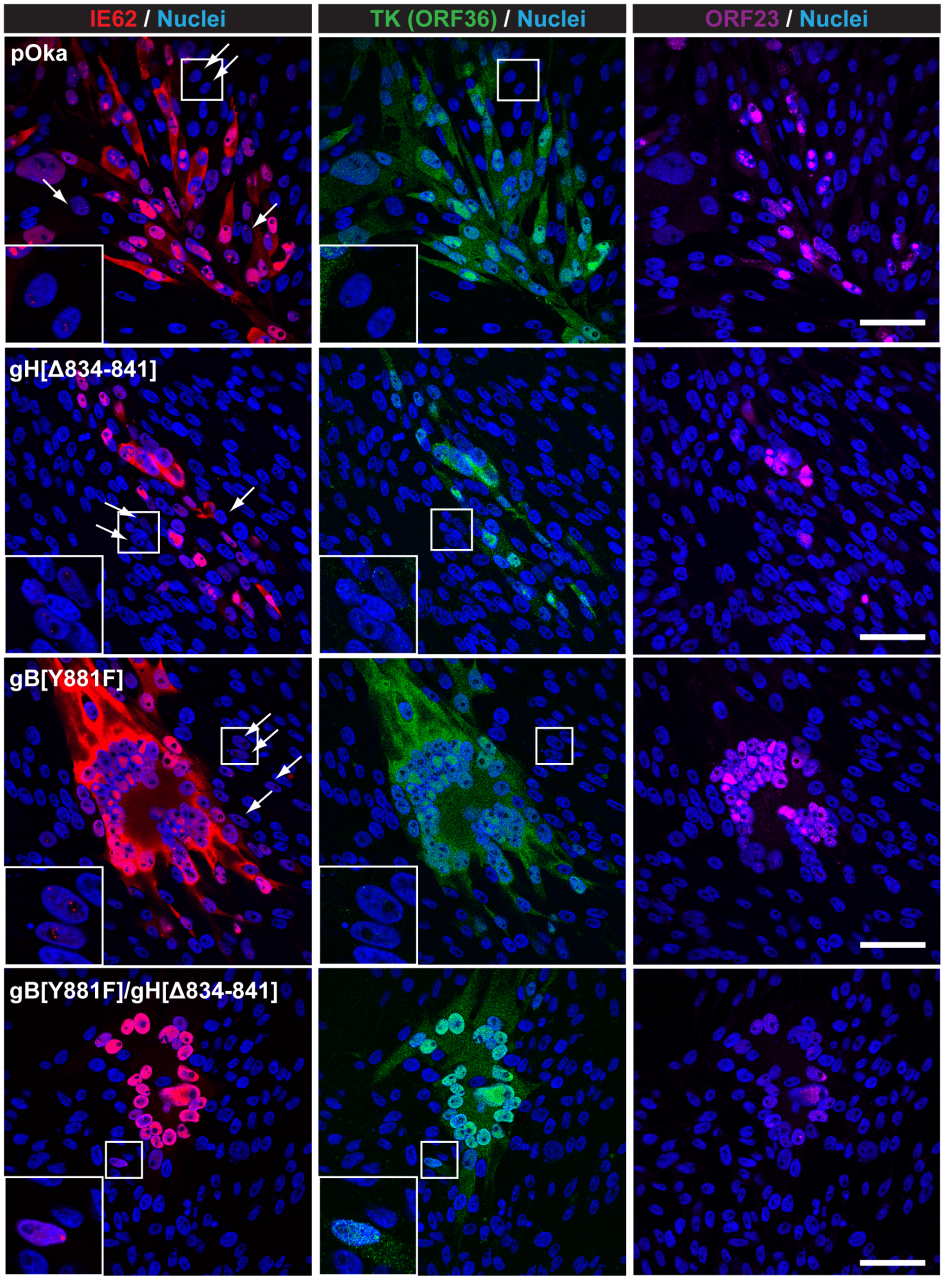
Dual mutant gB[Y881F]/gH[Δ834-841] BAC induces exaggerated syncytia formation. Confocal microscopy images of melanoma cells transfected with pOka-TK-GFP, pOka-TK-GFP-gH[Δ834-841], pOka-TK-GFP-gB[Y881F], and pOka-TK-GFP-gB[Y881F]/gH[Δ834-841] BACs at 72 hours post transfection, which also expressed a thymidine kinase (TK), ORF36, fusion protein tagged at the C terminus with green fluorescent protein (GFP). Cells were stained for immediate early proteins (IE62; red), capsid proteins (ORF23; violet), and nuclei (Hoechst 33342; blue). The white arrows indicate cells in the early stages of infection that have punctate staining of IE62 and little to no TK expression. Micrographs in the left bottom corner are magnified images of the cells boxed in white. The scale bars represent 50 µm.

To examine if removal of the fusion regulatory elements of both the gBcyt and gHcyt further exacerbated syncytia formation during infection, a BAC with amino acids 834-841 of gH deleted and the Y881 in the gBcyt ITIM substituted with phenylalanine was constructed using a pOka BAC that expressed ORF36, the thymidine kinase, tagged with EGFP at the C-terminus (pOka-TK-GFP). Infectious virus was not produced in melanoma cells transfected with the double mutant BAC, pOka-TK-GFP-gB[Y881F]/gH[Δ834-841]. In contrast, infectious virus was obtained from pOka-TK-GFP-gH[Δ834-841] and -gB[Y881F] BACs and melanoma cells infected with these viruses exhibited exaggerated syncytia phenotypes that were similar to their non TK-GFP counterparts. While infectious virus was not recovered from pOka-TK-GFP- gB[Y881F]/gH[Δ834-841], exaggerated syncytia formation occurred in BAC-transfected melanoma cells at 72 hours post transfection (Figure 10). To examine if the gB[Y881F]/gH[Δ834-841] mutations modified localization of immediate early proteins or capsid proteins, cells were stained for IE62 and capsid protein, ORF23, respectively. While accumulated nuclear localization of IE62 was observed for melanoma cells transfected with pOka-TK-GFP BAC and all three mutants, only pOka-TK-GFP, pOka-TK-GFP-gH[Δ834-841], and pOka-TK-GFP-gB[Y881F] had cytoplasmic localization of IE62, suggesting that the gB[Y881F]/gH[Δ834-841] mutations prevented the cell from entering the late stages of VZV infection. In addition, nuclei that had punctate IE62 expression were also observed in melanoma cells transfected with pOka-TK-GFP, pOka-TK-GFP-gH[Δ834-841], and pOka-TK-GFP- gB[Y881F] BACs consistent with early viral replication (Figure 10, white arrows). This pattern was not seen with melanoma cells transfected with pOka-TK-GFP-gB[Y881F]/gH[Δ834-841] BAC, indicating that the exaggerated syncytia formation either limited viral replication or spread. Capsid protein, ORF23, was detected in nuclei after transfection of all three mutants and pOka-TK-GFP, although levels were slightly reduced in cells transfected with pOka-TK-GFP- gB[Y881F]/gH[Δ834-841]. Syncytia formation was also more apparent at 72 hours post transfection, with pOka-TK-GFP-gB[Y881F] and -gB[Y881F]/gH[Δ834-841] having a rosette-like clustering of nuclei in the center of the syncytia compared to pOka-TK-GFP. In contrast, both pOka-TK-GFP-gH[Δ834-841] and pOka-TK-GFP exhibited smaller syncytia at 72 hours post transfection, suggesting that the exaggerated syncytia phenotype associated with pOka-TK-GFP-gH[Δ834-841] occurred later in infection while that induced by pOka-TK-GFP-gB[Y881F] either occurred earlier or was further enhanced (Figure 10).

**Figure 11:**
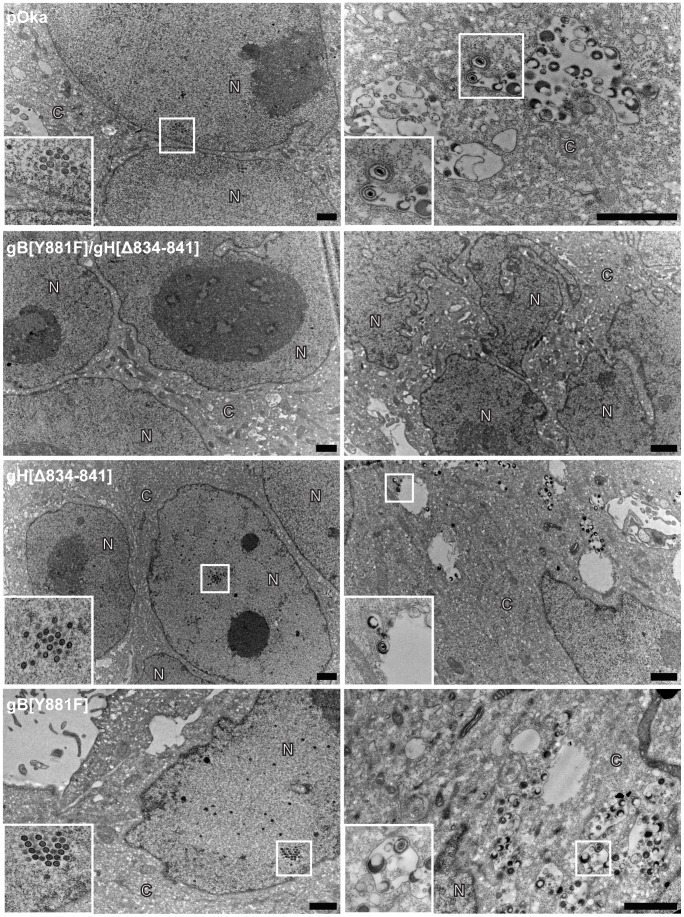
Viral particles are not observed in melanoma cells transfected with dual mutant gB[Y881F]/gH[Δ834-841] BAC. Electronmicrographs of FACS-enriched melanoma cells transfected with pOka-TK-GFP, pOka- TK-GFP-gH[Δ834-841], pOka-TK-GFP-gB[Y881F], and pOka-TK-GFP-gB[Y881F]/gH[Δ834-841] BACs at 72 hours post transfection. Images in the left and right column focus on the nuclei and cytoplasm of syncytium, respectively. The images in the bottom left corner represent magnified images of the areas boxed in white. The C and N denote cytoplasmic and nuclear space, respectively. The scale bars represents 1 µm.

To determine if the pOka-TK-GFP-gB[Y881F]/gH[Δ834-841] BAC was able to generate virus particles, melanoma cells transfected with the mutant BAC were examined at 72 hours post transfection by electron microscopy (Figure 11). Consistent with the confocal micrographs, syncytia were observed in melanoma cells transfected with pOka-TK-GFP, pOka-TK-GFP-gH[Δ834-841], pOka-TK-GFP-gB[Y881F], and pOka-TK-GFP-gB[Y881F]/gH[Δ834-841] BACs. In contrast to pOka, gH[Δ834-841], and gB[Y881F], nucleocapsids and virus particles were not observed in cells transfected with the pOka-TK-GFP-gB[Y881F]/gH[Δ834-841] BAC. This correlated with the reduced ORF23 expression and limited IE62 localization observed in the confocal micrographs. The failure of the pOka-TK-GFP-gB[Y881F]/gH[Δ834-841] BAC to generate viral particles while still being able to induce syncytia formation suggested that VZV-induced cell-cell fusion is the result of viral protein synthesis but does not require a full viral replication cycle. Furthermore, these data indicate that loss of both the gBcyt and gHcyt fusion regulatory elements generates an intracellular environment that limits viral production and spread.

### Deletion of the gHcyt significantly impairs VZV pathogenesis in skin

**Figure 12:**
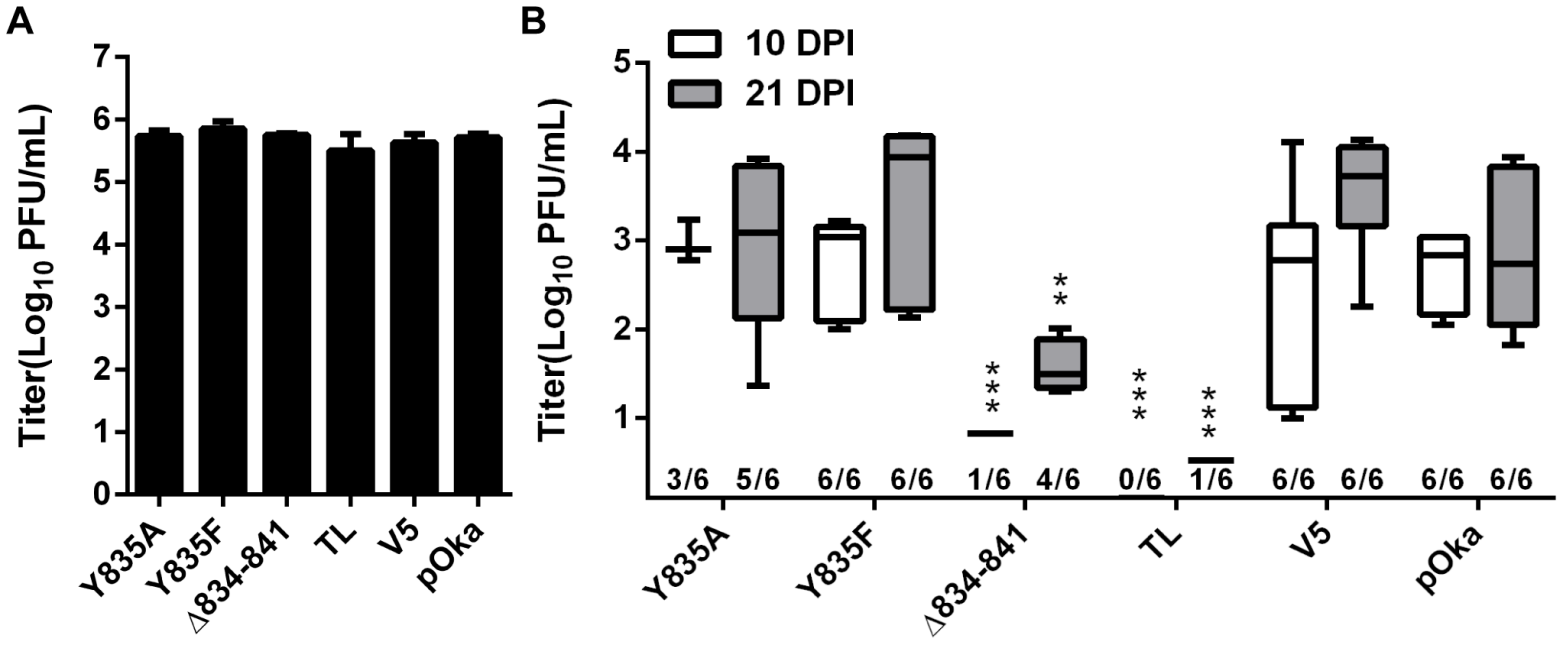
Truncation of the gHcyt restricts VZV pathogenesis in human skin xenografts. (A) Mean titers of inoculum of pOka, pOka-gH[Y835A], -gH[Y835F], -gH[V5], -gH[Δ834-841], and -gH[TL] viruses used for infection of human skin xenografts. Inoculum was titrated in triplicate. The error bars indicate the SEM. (B) Box and whiskers plot of skin xenograft infection at 10 (white) and 21 (grey) days post infection (dpi) titrated in triplicate on melanoma cells. The boxes indicate the 10–90% percentile with the solid bar inside the box representing the median and the whiskers indicating the minimum and maximum of all titers. The numbers along the x-axis represent the frequency of virus-positive xenografts per number inoculated for each virus. Statistical differences were determined by two-way ANOVA by comparing the titers for the gH mutants to either pOka at 10 or 21 dpi. (**P<0.05, ***P<0.001).

To determine effects of the gHcyt mutations and dysregulation of syncytia formation on VZV pathogenesis *in vivo*, human skin xenografts were infected with pOka, pOka-gH[Δ834-841], pOka-gH[Y835A], pOka-gH[Y835F], pOka-gH[V5], and pOka-gH[TL] viruses at similar inoculum titers (Figure 12A). At 10 dpi, viral titers from skin xenografts infected with pOka-gH[Y835A] and pOka-gH[Y835F] mutants were not significantly different from pOka, indicating that the ^835^YNKI^838^ motif had no role in skin pathogenesis. The pOka-gH[V5] virus also replicated at similar levels to pOka. In contrast, infectious virus was not recovered from any of the six xenografts infected with pOka-gH[TL] or from five of the six pOka-gH[Δ834-841]-infected xenografts; the titer of the sixth implant was <1 log_10_ (Figure 12B). Similar results were observed in xenografts recovered at 21 dpi after inoculation of pOka-gH[Y835A], pOka-gH[Y835F], and pOka-gH[V5] viruses, which had titers that were again comparable to pOka. pOka-gH[TL] virus was not isolated from five of the six xenografts with the sixth having a titer of <1 log_10_. The pOka-gH[Δ834-841] infected implants showed some limited levels of viral replication with four of the six implants having a mean titer of 1.5 log_10_, approximately 1.3 log_10_ lower than the mean titers of pOka-infected xenografts. Thus, the gHcyt played a critical role in VZV skin pathogenesis and was dependent on amino acids 834-841 of gH.

**Figure 13:**
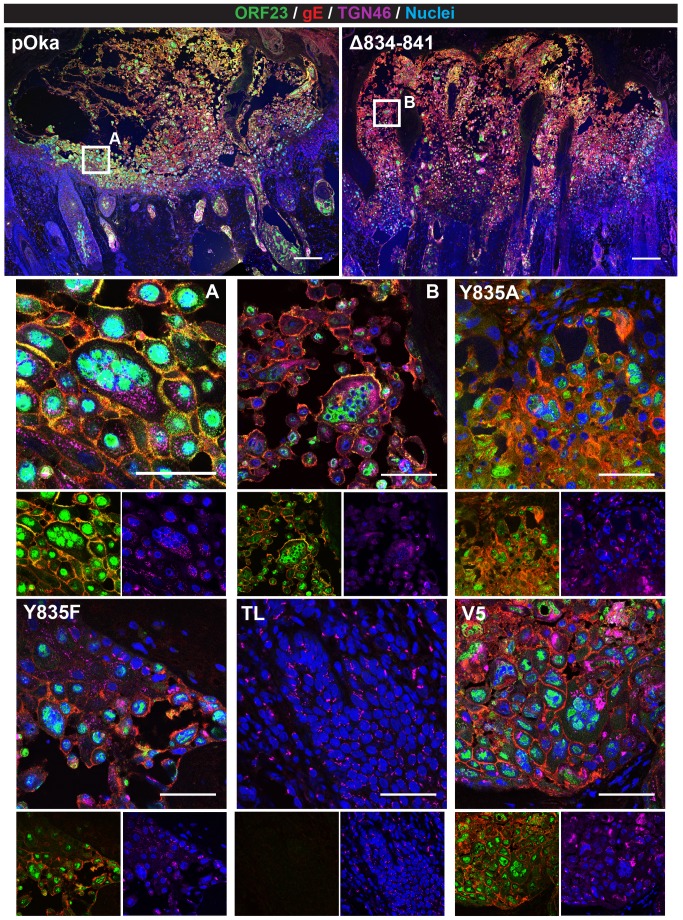
Pathogenesis of VZV with mutations in the gHcyt in human skin xenografts. Confocal micrographs of 5 µm sections of human skin xenografts with the highest titers infected with either pOka or pOka-gH[Δ834-841], -gH[Y835A], -gH[Y835F], -gH[TL], and -gH[V5] at 21 dpi. The sections were stained for capsids (ORF23; green), gE (red), TGN46 (violet), and nuclei (Hoechst 33342; blue). The scale bar for skin implants infected with pOka (top left) and gH[Δ834-841] (top right) represent 200 µm. White squares are shown in higher magnification immediately below (A = pOka, B = Δ834-841). Higher magnification images consist of a four-color merge (top), two color-ORF23 and gE merge (bottom left), and two color-TGN46 and nuclei merge (bottom right). Lesions were not detected for pOka-gH[TL]-infected xenografts. The scale bars for higher magnification images represent 50 µm.

To further examine VZV skin pathogenesis, confocal microscopy was performed on sections from xenografts at 21 dpi that yielded the highest viral titers. Infected cells were identified by ORF23 and gE expression, with the skin layers visualized by staining for TGN46 and nuclei (Figure 13). As previously reported [34], the lesion in the pOka-infected xenograft showed penetration and spread of the virus across the epidermal and basal layer into the dermal layer (Figure 13, top left). Infection was also visible in hair follicles and syncytia were identified scattered throughout the lesion (Figure 13, A). Similar to pOka, the single pOka-gH[Δ834-841]-infected xenograft that had a titer of 2 log_10_ also showed penetration and spread through all three layers of the skin (Figure 13, B). In addition, differences in syncytia morphology and size were not apparent between pOka and pOka-gH[Δ834-841]. Lesions from xenografts infected with pOka-gH[Y835A], pOka-gH[Y835F], and pOka[gH-V5] viruses as well as ORF23 and gE staining were all morphologically similar to pOka. Lesions were not identified in the single xenograft from which pOka-gH[TL] was recovered, which correlated with the extremely low viral titer (Figure 12B).

## Discussion

Syncytia formation is an inherent characteristic of VZV replication *in vitro* and *in vivo*. In this study, amino acids 834-841 of the VZV gH cytoplasmic domain were identified to be critical for the regulation of gB/gH-gL mediated cell-cell fusion with truncation of the domain causing exaggerated syncytia formation during infection. While this exaggerated syncytia formation only caused a moderate reduction of viral titers in cultured cells, skin pathogenesis was significantly impaired. This data and our prior studies show that this regulation of cell fusion is mediated by two separate mechanisms involving both the gHcyt and gBcyt, with loss of either resulting in limited viral replication and spread *in vivo*. Eliminating both blocks VZV replication completely. Thus, the gHcyt, along with the gBcyt, plays a critical role in skin pathogenesis through their shared capacity to maintain regulation of VZV-induced cell fusion.

### Skin pathogenesis and regulation of cell-cell fusion is mediated by the cytoplasmic domains of both gH and gB

This study elucidates the functional domains of gH and gB important for cell fusion and demonstrates that the regulation of syncytia formation is necessary to support effective skin pathogenesis. In the case of VZV, the current model of gB acting as the primary fusogen is supported by the requirement for gB as demonstrated by the inability of gH[TL] and gL in the absence of gB to induce the high levels of fusion observed with gB/gH[TL]-gL. Regulation of cell fusion by gH was determined to depend on the length of its cytoplasmic domain with truncations of the domain causing increased cell fusion, indicative of a loss in regulation. Deletions and substitutions of amino acids 834-841 of the gHcyt confirmed that the regulation was not dependent upon a unique motif or specific biochemical properties such as hydrophobicity. Given that direct protein interactions commonly depend upon the presence of a specific motif or domain, it is unlikely that the gHcyt functions by a direct interaction with either a host or viral protein [41]. However, while it might not interact directly, the gHcyt is proposed to operate as a gate keeper using its physical length to control access of functional domains within neighboring proteins.

The gB/gH-gL mediated cell-cell fusion is also regulated by the gBcyt ITIM which has the potential to be phosphorylated at Y881 [34]. VZV mutagenesis to substitute the tyrosine residue with a phenylalanine (gB[Y881F]), which prevents phosphorylation of the residue, resulted in exaggerated syncytia formation in melanoma cells and significantly reduced skin pathogenesis. In contrast to infected cells, the inability of the gB[Y881F] mutation to induce high levels of fusion when expressed with gH[WT] in the Cre reporter assay strongly supports the notion that the full length gHcyt has complex fusion regulatory properties that are apparent during VZV infection. While a direct interaction of gB and gH-gL has not been confirmed, cell fusion requires both to be present on the cell surface and it is also probable that these two transmembrane glycoproteins, and thus their cytoplasmic domains, are located within close proximity on cell membranes. As a gate keeper, the gHcyt could function as a regulator of phosphorylation of the ITIM of the gBcyt by controlling either kinase or phosphatase access to Y881 residue. Therefore, the absence of the gHcyt gate keeper function in the gH[TL] enables gB[Y881F] to readily enter a dysregulated state and enhance fusion. This dysregulated state was detrimental to the virus as melanoma cells transfected with pOka BAC containing the dual gB[Y881F]/gH[Δ834-841] mutation were unable to effectively establish a progressive infection, while retaining the capacity to induce exaggerated syncytia formation. These data confirm that losing syncytia regulation significantly limits viral replication and spread. The gBcyt might also contribute to regulation of the early steps of cell fusion, as demonstrated by the LL871/872AA substitution in the HSV-1 gBcyt initiating fusion significantly more rapidly than the wildtype, while having surface expression levels that were similar to the wildtype [42]. The VZV gBcyt also contains a similar dileucine motif (^904^LL^905^). Further investigation is needed to determine if the motif has a similar functional regulatory role in fusion initiation. Together, these data establish that VZV gB and gH participate in a complex regulatory system to limit syncytia formation.

### Regulation of cell-cell membrane fusion differs from virion envelope and cell membrane fusion

Regulation of fusion between the virion envelope and cell membrane during entry has been postulated to be different from cell-cell fusion [43]. Our observations support this concept because while truncating the gHcyt increased cell fusion when transfected with gB and gL *in vitro* and caused exaggerated syncytia formation when introduced into the virus, the morphology of the viral particles and their presence on cell surfaces was similar to pOka. Since regulation of cell-cell fusion also depends upon phosphorylation of the gBcyt ITIM, identical regulation for both virion envelope and cell fusion would require the presence of kinases and phosphatases with the capacity to alter tyrosine phosphorylation within the virion as well as in infected cells. While mass spectrometry studies have not been performed on extracellular VZV virions, studies of HSV-1 virions have not shown incorporation of phosphatases or tyrosine kinases [44]. It has also been noted that the lipid and protein composition of the viral envelope membrane would be expected to be different from the cell membrane, which could also change the regulation of fusion [45]. While further studies are necessary, our findings support differences in how VZV gH and gB contribute to the regulation of cell fusion and virion entry.

### Differences in the regulation of gB/gH-gL mediated cell fusion by the cytoplasmic domain of HSV and VZV gH

In contrast to VZV, syncytia formation is not a canonical characteristic of HSV-1 infection which suggests that the fusion machinery for HSV-1 controls cell fusion more tightly than VZV. While syncytial (syn) strains of HSV-1 have been identified with mutations in either gB (UL27), gK (UL53), UL20, or UL24 [46], [47], HSV syn viruses with mutations in the HSV gHcyt have not been reported. Regulation of syncytia formation by the HSV gHcyt has been demonstrated by the reduction of the syncytial phenotype in cells either infected with the HSV-1 gB[V855A] syn mutant virus when the gHcyt was truncated by nine amino acids or a gH null/gB[V855A] syn mutant virus was trans-complemented with a plasmid expressing the truncated gH [43], [48], [49]. This was also observed with *in vitro* fusion studies as expression of the syncytial gB[A885V] mutant with the truncated gH reduced its hyperfusion levels from 200% to 150% of wildtype fusion levels [50]. These findings suggest the V855A substitution in the HSV-1 gBcyt inhibited the regulatory control of the gBcyt over cell fusion that was then rescued by the truncation of the HSV gHcyt. This supports the model that cell fusion is jointly regulated by the gBcyt and gHcyt. *In vitro* cell fusion studies involving transient expression of HSV-1 glycoproteins have provided further evidence of the role of the gHcyt in fusion and its regulation. Cell fusion was inhibited when the HSV-1 gHcyt was either completely deleted and replaced with analogous domains from other glycoproteins or when both the gH transmembrane and cytoplasmic domains were substituted with a glycosylphosphatidylinositol (gpi)-addition and expressed with gB, gD, and gL [51] [52]. Similar to VZV gH, truncation of the final six amino acids of HSV-1 gHcyt increased levels of cell fusion by 50% relative to the wildtype gH when transfected with gB[WT], gD, and gL [50]. Whether inserting this gH mutation into the viral genome would produce a HSV syn mutant is not known. Also consistent with our observations about the VZV gHcyt, the length of the HSV-1 gHcyt might be important for its contribution to regulation of cell fusion [53]. An insertion of five amino acids at 824L, increasing the length of the gHcyt from 14 to 20 amino acids, inhibited cell fusion in the presence of gB[WT]. Nevertheless, in the context of infection, VZV and HSV regulation of cell fusion and syncytia formation differ since truncation of VZV gH caused exaggerated syncytia formation, while HSV gH truncation reduced syncytia formation by the gB syn mutant. Thus, the VZV gB/gH-gL core fusion machinery has characteristics that change its fusogenicity capacity compared to HSV.

While the VZV gHcyt has been predicted to be 18 amino acids in length, the HSV gHcyt is only 14 amino acids long and no motifs or sequences are conserved between the two homologues. Furthermore, induction of cell fusion by HSV *in vitro* requires not only gB/gH-gL, but also gD. These differences in sequence length and requirements for *in vitro* cell fusion make it challenging to compare the processes of HSV-1 and VZV cell fusion. However, it is apparent that the gHcyt domains of both viruses perform a significant function in cell fusion regulation which is linked to the regulation of their gB counterparts.

### The gHcyt YNKI motif does not have a functional role in syncytia formation

The gH ^835^YNKI^838^ motif does not mediate gB/gH-gL cell-cell fusion *in vitro*, virus-induced syncytia formation, and spread in cultured cells or skin pathogenesis. Disruption of the motif also did not alter gH expression or trafficking in melanoma cells that were transiently expressing gH-gL or infected with recombinant mutant VZV. These results are in contrast to an earlier report which indicated that the ^835^YNKI^838^ motif was a functional endocytosis motif based on effects of an identical tyrosine to alanine substitution examined by confocal microscopy and using a biotin endocytosis assay. While confocal microscopy was used to examine endocytosis in both studies, the first observations were based upon infecting HeLa cells with recombinant vaccinia virus (VV) expressing the T7 polymerase and co-transfecting with pTM1 plasmids, a T7-driven vector, expressing gH and gL [29]. Given that VV can induce significant changes in the cytoskeletal network of host cells [54] and the cell proteome [55], the modified intracellular environment and the additional VV proteins might have contributed to the inhibition of endocytosis of gH[Y835A]. In addition, we have shown that gH-gL co-expression to be insufficient for cell-cell fusion [27], [34] in contrast to a previous report [56]. HeLa cells infected with VV expressing gH with the Y835A substitution and gL showed increased syncytia size, which might also be explained by VV effects [33]. Vaccinia virus expresses its own acid induced membrane fusion protein on the surface of the infected cell [57] and regulates actin polymerization in the host cell, which might prime the cell to be more fusogenic. Our approach to examine the ^835^YNKI^838^ motif more accurately models the function and activity of gH since the virus-free transfection system does not have additional molecules that could potentially affect gH trafficking. Furthermore, gH was also examined under the context of infection, which allows for usual expression of other VZV proteins that could facilitate gH trafficking in infected cells by mechanisms other than the ^835^YNKI^838^ motif.

### Enhanced syncytia formation limits skin pathogenesis

**Figure 14:**
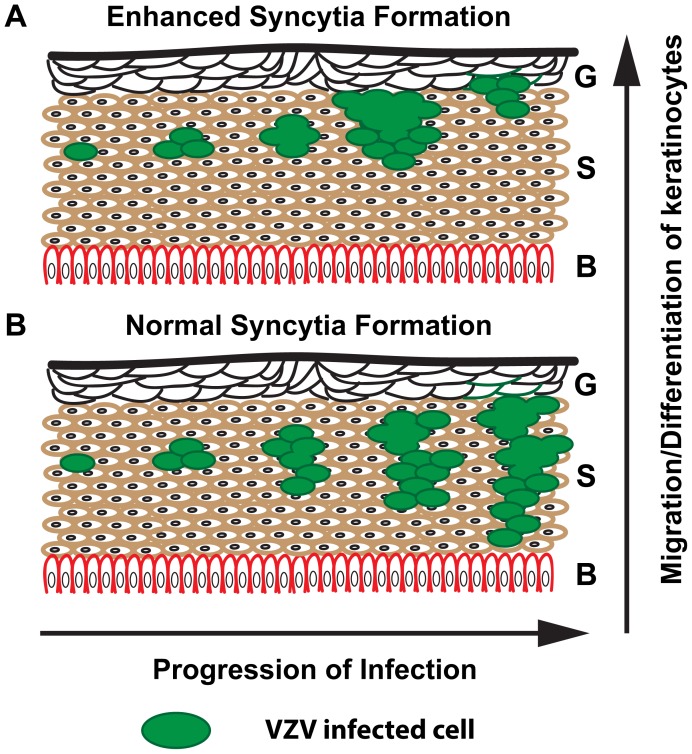
Proposed model for limited skin pathogenesis of VZV caused by exaggerated syncytia formation. Schematic of human skin with the three main layers denoted: G = Granular, S = Spinous, and B = Basal. (A) Exaggerated syncytia formation by pOka-gH[Δ834-841] and pOka-gH[TL] limits the virus from being able to spread effectively and penetrate the skin, causing the lesion to remain near the surface. (B) In contrast, normal syncytia formation by pOka allows the virus to easily spread and penetrate the basal layer allowing for expansion of the lesion.

The truncation of the VZV gHcyt increased syncytia formation and significantly reduced skin pathogenesis. A similar phenotype was observed with the gBcyt ITIM mutation indicating that exaggerated syncytia formation limits the ability of VZV to spread effectively in the skin. This demonstrates a direct link between skin pathogenesis and cell-cell fusion. The epidermis consists of three main layers, with the granular layer facing the surface, followed by the spinous layer and the inner basal layer. The epidermis is constantly in flux due to continued cell shedding that is quickly replaced by differentiated keratinocytes [58]. Exaggerated syncytia formation is proposed to limit the ability of the virus to effectively penetrate the skin, causing the lesion to remain near the surface (Figure 14A). If the rate of penetration is less than the rate of differentiation and migration of keratinocytes from the basal layer, then the epidermis would drive the infected cells out through the granular and spinous layers, preventing spread to deeper layers of the spinous and basal layer. When syncytia formation is regulated appropriately as in pOka-infected tissue, the virus is able to effectively spread and penetrate the skin creating the typical cutaneous lesions (Figure 14B). While this model requires further study, the present study demonstrates that tight regulation of syncytia formation through complementary functions of the cytoplasmic domains of gB and gH is critical for VZV pathogenesis in skin.

## Supporting Information

Figure S1 **Hydrophobic residues within the predicted cytoplasmic domain of gH homologues of alphaherpesviruses.** Predicted cytoplasmic domains of gH homologues of alphaherpesviruses with hydrophobic residues (Leucine = L, Valine = V, Isoleucine = I, Phenylalanine = F) shaded in black. Residues shaded in grey were predicted by TOPCONS to be within the transmembrane domain.
(TIF)

Figure S2 ***In vitro*** **intracellular localization and endocytosis of gH in melanoma cells are not affected by the hydrophobicity of the gHcyt.** (A) Hydrophobicity plot of gH[TL], gH[WT], gH[824(IV)^4^], and gH[824(QN)^4^] from residues 825 to 840. Values were calculated with ProtScale (http://web.expasy.org/cgi-bin/protscale/protscale.pl) using Kyte & Doolittle amino acid scale values with values greater than zero indicating residues with hydrophobic side chains [59]. (B) Confocal microscopy images of melanoma cells transiently expressing gH[TL], gH[WT], gH[824(IV)^4^], or gH[824(QN)^4^] with gL at 24 hours post transfection. Cells were stained for gH (red), early endosome antigen (EEA1; green), trans-Golgi network (TGN46; green), and nuclei (Hoechst 33342; blue). White arrows indicate colocalization of EEA1 and gH, which highlight representative endocytic vesicles containing gH. The scale bars represent 20 µm.
(TIF)

Figure S3 **Substitution of amino acids 834-841 affects cell surface expression of gH.** Cell surface expression of gH on CHO cells transfected with vectors expressing gH constructs and gL. VZV gH was detected with SG3 antibody (anti-gH) in nonpermeabilized cells using flow cytometry at 24 hours post transfection. The gH mutants gH[TL], gH[V5], gH[cMyc], gH[834StopV5], gH[824(IV)^4^], gH[824(QN)^4^], gH[Y835A], gH[Y835F], gH[Δ834-841], gH[Δ836-841], gH[Δ838-841], and gH[Δ840-841] (dotted line) were compared to their corresponding gH[WT] control (positive control, solid line), and either (A) gH[WT] without gL or (B) gH[TL] without gL (negative control, shaded). Each representative histogram shows the frequency of counts compared to the intensity of Alexa Fluor 488 used to detect gH. Black (mutant) and blue (positive control) numbers represent the percentage of cells with fluorescence greater than their corresponding negative control. Red numbers represent the cell surface levels of gH mutant expression normalized to their respective positive control, which was used to calculate the values in Figure 3.
(TIF)

Figure S4 **Truncation of the gHcyt results in syncytium with increased number of nuclei compared to syncytium of pOka-gH[Y835A] and pOka.** Dot plot of number of nuclei per syncytium of 15 randomly selected syncytium induced during infection of melanoma cells by pOka-gH[Δ834-841], pOka-gH[Y835A], and pOka at 36 hpi. Each circle represents a single syncytium. Bar indicates the mean. (***P<0.001).
(TIF)

Figure S5 **Substitution of amino acids 834-841 of gH with Y835A, Y835F, and V5 does not affect syncytia formation during infection of melanoma cells.** Merged phase contrast and fluorescence microscopy images of melanoma cells infected with pOka, pOka-gH[TL], pOka-gH[V5], pOka-gH[Y835A], and pOka-gH[Y835F] viruses at 36 and 48 hpi. The nuclei were stained with Hoechst 33342 (blue). Visually detectable syncytia are outlined in red with white arrows indicating extended cytoplasm of VZV-induced syncytia. The scale bars represent 200 µm.
(TIF)

Table S1 **Primers used for VZV gH mutagenesis.**
(DOC)
