# Supplementary figures and images for: The Cytoplasmic Domain of Varicella-Zoster Virus Glycoprotein H Regulates Syncytia Formation and Skin Pathogenesis
Source: PLoS Pathog. 2014 May 29;10(5):e1004173. doi: 10.1371/journal.ppat.1004173 (PMC4038623; doi:10.1371/journal.ppat.1004173)

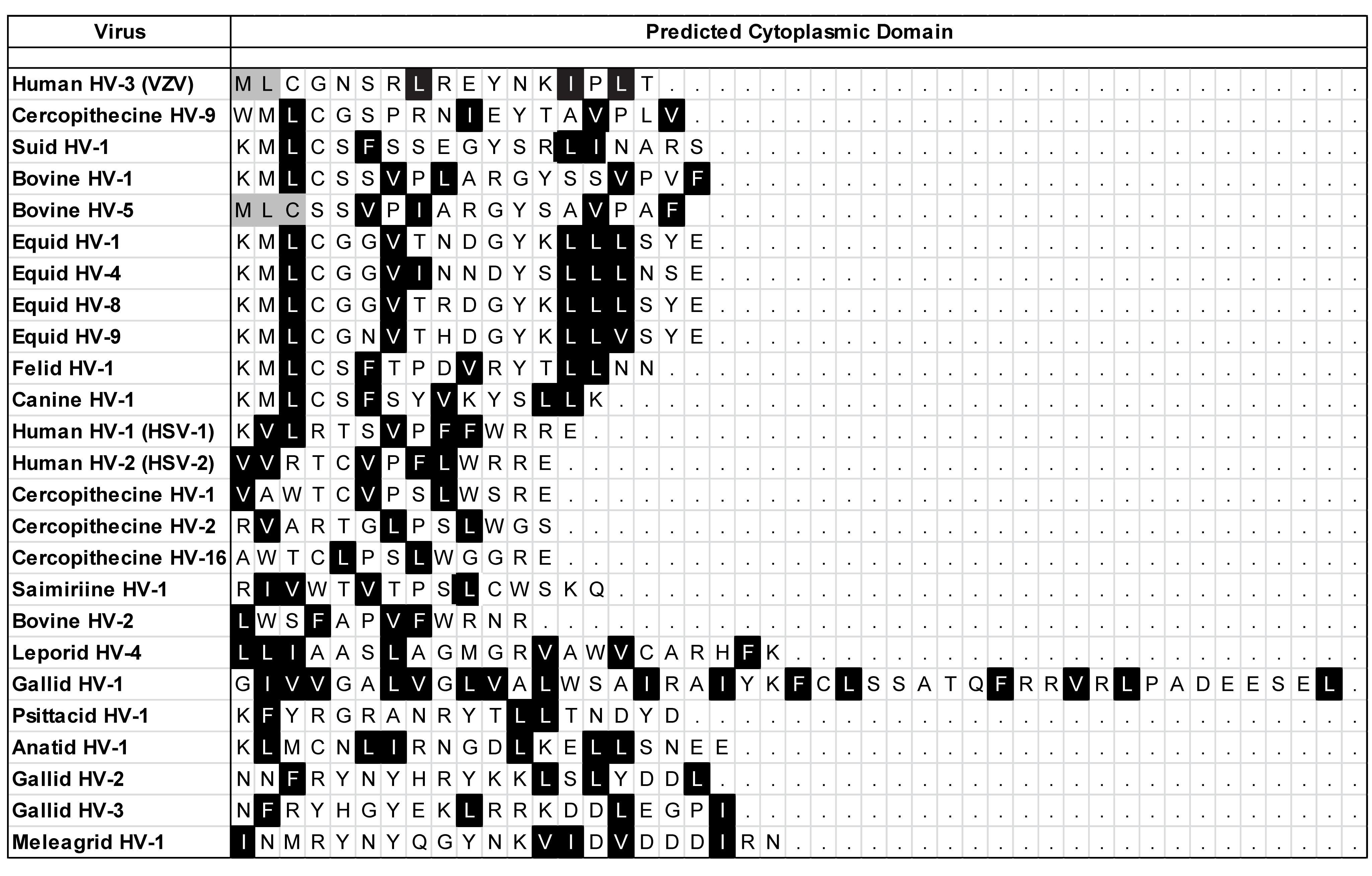

Supplement: Figure S1 — Hydrophobic residues within the predicted cytoplasmic domain of gH homologues of alphaherpesviruses. Predicted cytoplasmic domains of gH homologues of alphaherpesviruses with hydrophobic residues (Leucine = L, Valine = V, Isoleucine = I, Phenylalanine = F) shaded in black. Residues shaded in grey were predicted by TOPCONS to be within the transmembrane domain. (TIF) [file ppat.1004173.s001.tif]

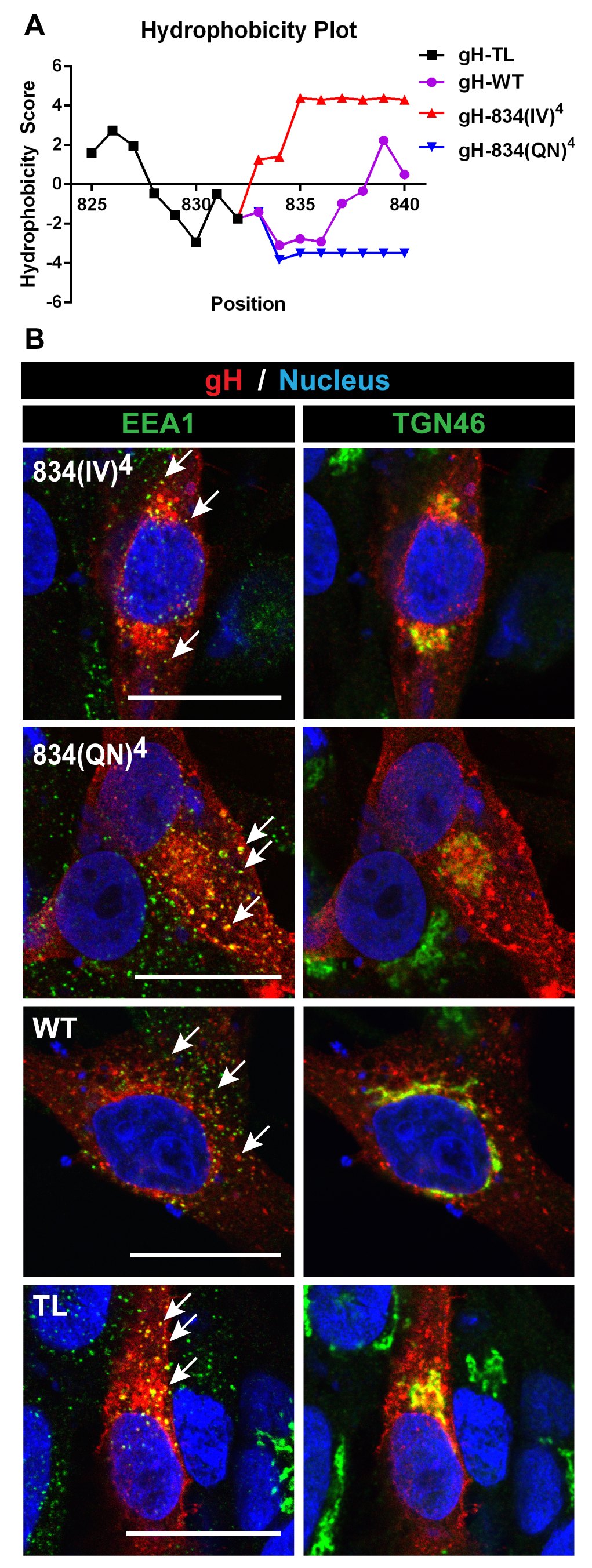

Supplement: Figure S2 — In vitro intracellular localization and endocytosis of gH in melanoma cells are not affected by the hydrophobicity of the gHcyt. (A) Hydrophobicity plot of gH[TL], gH[WT], gH[824(IV)4], and gH[824(QN)4] from residues 825 to 840. Values were calculated with ProtScale (http://web.expasy.org/cgi-bin/protscale/protscale.pl) using Kyte & Doolittle amino acid scale values with values greater than zero indicating residues with hydrophobic side chains [59]. (B) Confocal microscopy images of melanoma cells transiently expressing gH[TL], gH[WT], gH[824(IV)4], or gH[824(QN)4] with gL at 24 hours post transfection. Cells were stained for gH (red), early endosome antigen (EEA1; green), trans-Golgi network (TGN46; green), and nuclei (Hoechst 33342; blue). White arrows indicate colocalization of EEA1 and gH, which highlight representative endocytic vesicles containing gH. The scale bars represent 20 µm. (TIF) [file ppat.1004173.s002.tif]

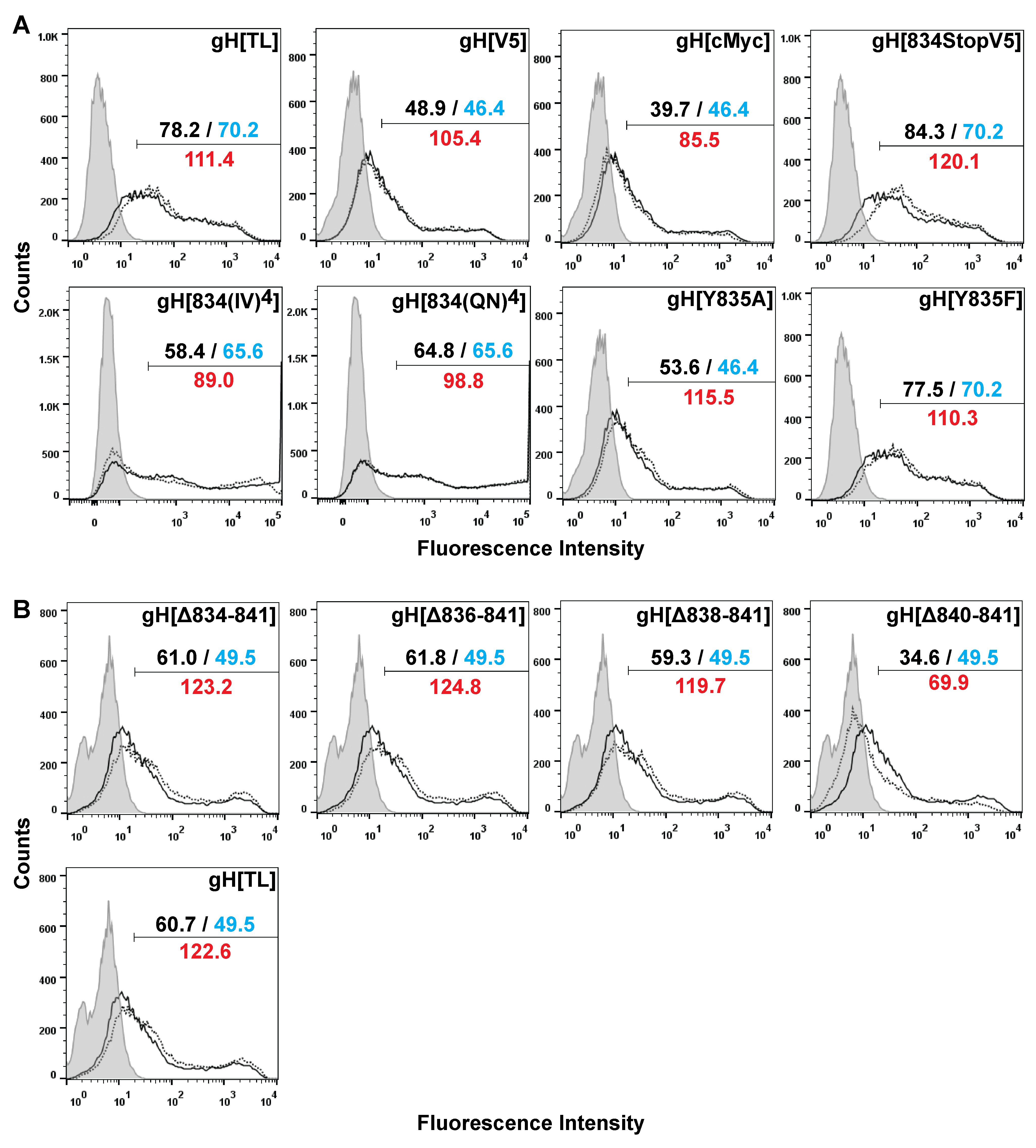

Supplement: Figure S3 — Substitution of amino acids 834-841 affects cell surface expression of gH. Cell surface expression of gH on CHO cells transfected with vectors expressing gH constructs and gL. VZV gH was detected with SG3 antibody (anti-gH) in nonpermeabilized cells using flow cytometry at 24 hours post transfection. The gH mutants gH[TL], gH[V5], gH[cMyc], gH[834StopV5], gH[824(IV)4], gH[824(QN)4], gH[Y835A], gH[Y835F], gH[Δ834-841], gH[Δ836-841], gH[Δ838-841], and gH[Δ840-841] (dotted line) were compared to their corresponding gH[WT] control (positive control, solid line), and either (A) gH[WT] without gL or (B) gH[TL] without gL (negative control, shaded). Each representative histogram shows the frequency of counts compared to the intensity of Alexa Fluor 488 used to detect gH. Black (mutant) and blue (positive control) numbers represent the percentage of cells with fluorescence greater than their corresponding negative control. Red numbers represent the cell surface levels of gH mutant expression normalized to their respective positive control, which was used to calculate the values in Figure 3. (TIF) [file ppat.1004173.s003.tif]

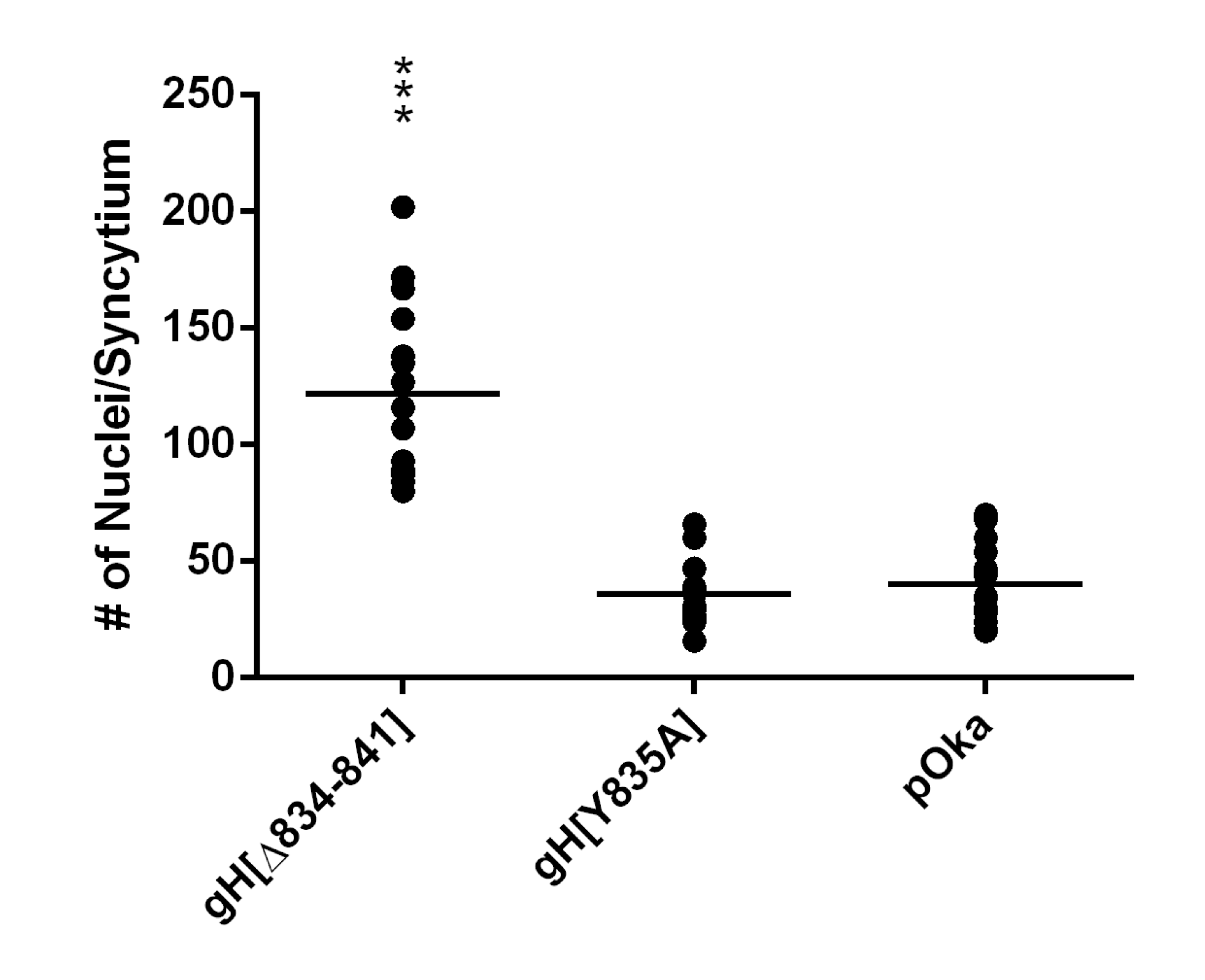

Supplement: Figure S4 — Truncation of the gHcyt results in syncytium with increased number of nuclei compared to syncytium of pOka-gH[Y835A] and pOka. Dot plot of number of nuclei per syncytium of 15 randomly selected syncytium induced during infection of melanoma cells by pOka-gH[Δ834-841], pOka-gH[Y835A], and pOka at 36 hpi. Each circle represents a single syncytium. Bar indicates the mean. (***P<0.001). (TIF) [file ppat.1004173.s004.tif]

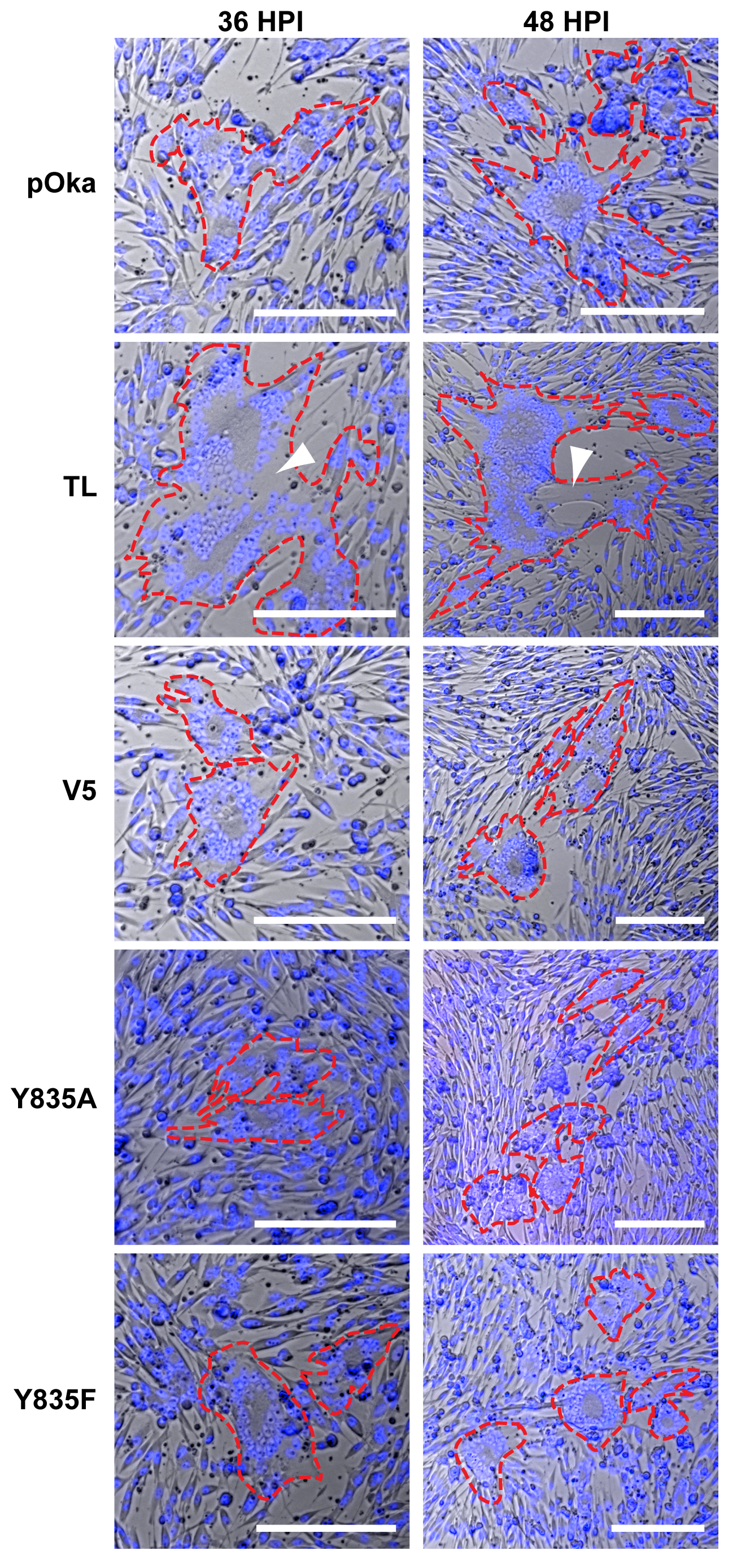

Supplement: Figure S5 — Substitution of amino acids 834-841 of gH with Y835A, Y835F, and V5 does not affect syncytia formation during infection of melanoma cells. Merged phase contrast and fluorescence microscopy images of melanoma cells infected with pOka, pOka-gH[TL], pOka-gH[V5], pOka-gH[Y835A], and pOka-gH[Y835F] viruses at 36 and 48 hpi. The nuclei were stained with Hoechst 33342 (blue). Visually detectable syncytia are outlined in red with white arrows indicating extended cytoplasm of VZV-induced syncytia. The scale bars represent 200 µm. (TIF) [file ppat.1004173.s005.tif]
