# Supplementary material for: The Cytoplasmic Domain of Varicella-Zoster Virus Glycoprotein H Regulates Syncytia Formation and Skin Pathogenesis
Source: PLoS Pathog. 2014 May 29;10(5):e1004173. doi: 10.1371/journal.ppat.1004173 (PMC4038623; doi:10.1371/journal.ppat.1004173)
Supplement: Table S1 — Primers used for VZV gH mutagenesis. (DOC) [file ppat.1004173.s006.doc]

| **Primer Name** | **Sequence** |
| --- | --- |
| gH[37]1697AccI-F | TACAATACCAAACGTATACAGTCC |
| [Phos]4119-4139 | CGAAGGCGGGAATTTCCACAT |
| [Phos]pME18s-gHd834-841 | ATAAGCGGCCGCTAGACTAGTCTAG |
| pME18s-KPN1 | ATTAATTCGAGCTCGGTACCCG |
| [Phos]ANKI | AGAAGCTAATAAAATACCTCTGACAT |
| [Phos]FNKI | AGAATTCAATAAAATACCTCTGACAT |
| [Phos]gH834-stop-V5 | ATAAGGTAAGCCTATCCCTAACCC |
| [Phos]gH-c-MYC | AGAACAGAAGCTGATCTCAGAGGAAGACCTCTAATAAGCGGCCGCTAGACTAGTCTAG |
| ORF37[gH]2495-2499V5 | AAAGCGGCCGCTTATTACGTAGAATCGAGACCGAGGAGAGGGTTAGGGATAGGCTTACCTCCGAAGGCGGGAATTTCCACATAAC |
| [Phos]gH-836-841 | ATATTCTCGAAGGCGGGAATTTCCA |
| [Phos]gH-838-841 | TTTATTATATTCTCGAAGGCGGGAA |
| [Phos]gH-840-841 | AGGTATTTTATTATATTCTCGAAGG |
| [Phos]pME18s-gHstop | TAAGCGGCCGCTAGACTAGTCTAGA |
| BsrG1-pCR4 | ACTCTGTACATCCACAAACAGACG |
| [Phos]gH-Kan-834-stop | ATAATATAATAAAATACCTCTGACATAAAAAAC |
| [Phos]V5-stop | TTACGTAGAATCGAGACCGAGG |
| [Phos]gH-Kan4168-4191 | AAAACATGTATAATAAAAAGTCAC |
| [Phos]gH834IVIVIVIV | AATCGTCATCGTCATCGTCATCGTCTAAGCGGCCGCTAGACTAGTCTAG |
| [Phos]gH834QNQNQNQN | ACAGAACCAGAACCAGAACCAGAACTAAGCGGCCGCTAGACTAGTCTAG |
| {37}F65680-65700 | ACAACACTTCCTAAATATACC |
| {37}R68697-68717 | AACGGCGCTGTGCGTCTATGC |
| P1 (mini-F) | TTAACTCAGTTTCAATACGGTGCAG |
| P2 (mini-F) | TGGGGTTTCTTCTCAGGCTATC |
| P3 (cat) | AGGCATTTCAGTCAGTTGCTC |
| P4 (cat) | TGCCACTCATCGCAGTACTG |
| gB003 | AATGACACGTACATGGTTGCC |
| gB008 | AGAGTTTTGAAGTATAATGCG |
| {37}F1061-1081 | CGGATCAACATGACATAAACG |
| AfL 15678L | AACCGTATTCTGAGAAACAGCC |
| 5’TK-EGFP | AATGCGCATGGAATATATTGAAAGAGCTTGTTAATGCCGTTCAGGACAACACTTCCGTGAGCAAGGGCGAGGAGCTGTTC |
| 3’TK-EGFP | AAAGATATCATCTTTTTACTGGTACATACGTAAATACTAGGTATATTTACTTGTACAGCTCGTCCATGCCGAG |
